# Targeting the cannabinoid system to counteract the deleterious effects of stress in Alzheimer’s disease

**DOI:** 10.3389/fnagi.2022.949361

**Published:** 2022-10-04

**Authors:** Ronnie D. Shade, Jennifer A. Ross, Elisabeth J. Van Bockstaele

**Affiliations:** ^1^Philadelphia College of Osteopathic Medicine, Philadelphia, PA, United States; ^2^Department of Pharmacology and Physiology, College of Medicine, Drexel University, Philadelphia, PA, United States

**Keywords:** stress, locus coeruleus (LC), corticotropin releasing factor (CRF), hypothalamic-pitu itary-adrenal (HPA) axis, Alzheimer’s disease (AD)

## Abstract

*Alzheimer’s disease* is a progressive neurodegenerative disorder characterized histologically in postmortem human brains by the presence of dense protein accumulations known as amyloid plaques and tau tangles. Plaques and tangles develop over decades of aberrant protein processing, post-translational modification, and misfolding throughout an individual’s lifetime. We present a foundation of evidence from the literature that suggests chronic stress is associated with increased disease severity in Alzheimer’s patient populations. Taken together with preclinical evidence that chronic stress signaling can precipitate cellular distress, we argue that chronic psychological stress renders select circuits more vulnerable to amyloid- and tau- related abnormalities. We discuss the ongoing investigation of systemic and cellular processes that maintain the integrity of protein homeostasis in health and in degenerative conditions such as Alzheimer’s disease that have revealed multiple potential therapeutic avenues. For example, the endogenous cannabinoid system traverses the central and peripheral neural systems while simultaneously exerting anti-inflammatory influence over the immune response in the brain and throughout the body. Moreover, the cannabinoid system converges on several stress-integrative neuronal circuits and critical regions of the hypothalamic-pituitary-adrenal axis, with the capacity to dampen responses to psychological and cellular stress. Targeting the cannabinoid system by influencing endogenous processes or exogenously stimulating cannabinoid receptors with natural or synthetic cannabis compounds has been identified as a promising route for Alzheimer’s Disease intervention. We build on our foundational framework focusing on the significance of chronic psychological and cellular stress on the development of Alzheimer’s neuropathology by integrating literature on cannabinoid function and dysfunction within Alzheimer’s Disease and conclude with remarks on optimal strategies for treatment potential.

## Introduction

Allostasis is the process of the body managing stress through an intricate system involving central and peripheral nervous systems. Under various levels of physiological or psychological stress, known as allostatic load, the body elicits the hypothalamic-pituitary-adrenal (HPA) axis, prefrontal-locus coeruleus circuitry, and limbic system to maintain homeostasis. However, these advantageous mechanisms can become maladaptive under chronic stress, known as allostatic overload (McEwen, 2004; McEwen and Gianaros, 2011). Allostatic overload renders the body incapable of terminating the stress response. This eventually compromises the formidability of the body resulting in pathophysiology through inflammatory, cellular, and neuronal remodeling (McEwen, 2004; McEwen and Gianaros, 2011).

The current review focuses on the role of allostatic overload and the associated stress neurocircuitry in Alzheimer’s Disease (AD). AD is a progressive neurodegenerative disorder marked by cognitive deterioration and dysautonomia. It is the most common cause of dementia and affects approximately 1 in 9 people in populations 65 years of age and older (Nestor et al., 2004; Rajan et al., 2021). Typically, the first clinical sign is short-term memory loss (Godbolt et al., 2004; Hodges, 2006). As AD manifests over time, an individual will exhibit other signs of allostatic overload including delusions, depression, irritability, praxis, dysautonomia, endocrine dysfunction, and dementia (Ishii and Iadecola, 2015). Commonly, AD is diagnosed with the Diagnostic and Statistical Manual of Mental Disorders 5 (DSM-5) criteria for major neurocognitive disorder due to AD. The criteria include a gradual and progressive onset, cognitive decline in at least one domain, compromission of activities of daily living due to cognitive impairment, exclusion of cognitive deficits due to secondary etiology, and family history or genetic factors (Jack et al., 2011). In the past, the National Institute of Neurological and Communicative Diseases and Stroke/Alzheimer’s Disease and Related Disorders Association (NINCDS-ADRDA) criteria revolved around a clinical and pathological approach including clinical history, physical exam, neuropsychological testing, laboratory testing, and brain imaging (McKhann et al., 1984). Now, the NINCDS-ADRDA is refined to a new and broader criterion known as the National Institute of Aging/Alzheimer’s Association (NIA-AA) which is used to consider the clinical framework with biomarkers. The NIA-AA criteria encompasses preclinical AD, mild cognitive impairment secondary to AD, and dementia secondary to AD (Jack et al., 2011). Recently these criteria have been updated to reflect biological indices of disease that include the biomarkers β amyloid deposition (A), pathologic tau (T) and neurodegeneration (N), collectively referred to as ATN (Jack et al., 2018). A definitive AD diagnosis requires the presence of neurofibrillary tangles and amyloid plaques in brain autopsy (Mirra et al., 1991), thus the ATN research-based framework of biomarkers emphasizes the proteinopathy that distinguish AD from other neurological diseases (Jack et al., 2018).

### Disease heterogeneity: Emerging evidence and treatment implications

AD is generally considered an age-dependent disease whose patient population can broadly be divided into those that begin to exhibit symptoms before the age of 65, and those that start to experience symptoms at the age of 65 or older. Sporadic, or late onset AD (LOAD) occurs in individuals at least 65 years of age and is most associated with the apolipoprotein E (APOE) which is a predominant genetic biomarker risk factor (Schmidt et al., 2014). It is thought that lifestyle factors such as diet, exercise, and stress levels are significant factors in the development and progression of LOAD. Thus, LOAD may be considered an expression of allostatic overload that results from prolonged physiological and/or psychological stress over the course of a lifetime. This is contrasted with familial AD which develops earlier in life (< 65 years of age) and may be considered an expression of allostatic overload that results from extreme cellular stress. It is characterized by mutations in the genes for amyloid precursor protein (APP), presenilin 1 (PSEN1), and presenilin 2 (PSEN2). These familial AD-associated genes are responsible for the accelerated production and aggregation of amyloid proteins, resulting in about 5% of the total AD cases (Krstic and Knuesel, 2013). Regardless of early or late onset, suspected AD is confirmed by autopsy of human postmortem brain tissue. On a cellular level, allostatic overload is evident as abnormal protein aggregates resulting from cellular dysfunction.

Our understanding of clinical AD and the pathophysiology that underlies cognitive impairment continues to evolve as we conduct large-scale studies on AD patients. Recent efforts to discover a disease-modifying therapeutic have turned the focus of research toward the earliest, prodromal stages of disease. Combined with more in-depth research approaches, AD patient populations have come to be understood clinically and pathologically as much more heterogeneous than originally conceived. For example, positron emission tomography (PET) scanning studies using neuroimaging tracer compounds bound to amyloid-β (Aβ) deposits revealed amyloid deposition in 20–30% individuals without cognitive impairment (Rodrigue et al., 2009). Moreover, some asymptomatic AD individuals can deviate from the down spiraling of cognitive function mirrored by its age-dependent disease course. Researchers propose that there is a subtype of early onset of AD and a heterogeneity within asymptomatic AD individuals that are immune from clinical fatal outcomes although possessing neuropathology (Driscoll and Troncoso, 2011).

The heterogeneity of the AD patient population, particularly in early stages of disease, is further illustrated in a subset of the AD academic literature focused on the behavioral and psychological symptoms of dementia (BPSD) as well as other non-memory symptoms (McKeith and Cummings, 2005). One study found that dysfunction in judgment and problem solving, language, and visuospatial abilities were the first symptoms in the younger AD age presentation (Barnes et al., 2015). These findings were supported by another study reporting that non-memory cognition syndromes are recognized in the early onset of AD before age 65 (Mendez, 2012). This presents a reckoning of the predated, yet commonly used diagnosis of AD based on the NINCDS-ADRDA criteria to incorporate earlier onset AD symptoms without being limited to ruling-in dementia during the first step approach (McKhann et al., 1984; Dubois et al., 2007; Sutphen et al., 2014; Cece and Shifu, 2015). Moreover, neuropsychiatric assessments are thought to play a crucial role in accurately diagnosing and treating symptoms of dementia in diverse patient populations. Based on psychiatric evaluation alone, three behavioral subtypes of AD have been identified: those with low or absent behavioral abnormalities, those with clear symptoms of psychosis, and those with a persistent mood disorder (McKeith and Cummings, 2005).

Importantly, a culmination of neuroimaging and genomic evidence suggests significant neurobiological differences distinguish BPSD patients from those that do not exhibit psychiatric symptoms (Klimek et al., 1997; Ordway et al., 1997, 2003; McCulley et al., 2004; Szot et al., 2006; Borroni et al., 2009; Grunblatt et al., 2009; Combarros et al., 2010). The exploration of key modulators, receptors, and neuronal circuits in the pathogenesis of AD is an evolving field in targeting effective therapy. The continued failure of potential therapies in clinical and preclinical trials highlights the importance of not only understanding the clinical heterogeneity of AD, but also in concentrating our research efforts in delineating the neurobiological basis for these differences. For example, treatment aimed at dampening the pro-inflammatory effects of AD were demonstrated to be not only dependent upon the pathogenic stage of AD but also region specific. Thus, the diversity of cellular phenotypes and responses within different brain regions is significant in effectively treating cognitive abnormalities (Lopez-Gonzalez et al., 2015). In the coming sections, we put forth the hypothesis that chronic physiological or psychological stress, an expression of allostatic overload, renders a sub-population of individuals more vulnerable to stress-related disorders such as AD. Further, we argue that the neurological patterns that accompany chronic stress may be used as phenotypic markers for an AD patient subpopulation that may be more responsive to certain types of treatment. Specifically, we discuss the therapeutic potential of targeting the endogenous cannabinoid system to counteract the deleterious effects of stress at early stages of disease.

## Overview of Alzheimer’s disease neuropathology and stress-integrative circuitry

### Overview of Alzheimer’s disease neuropathology

Aβ plaques are one of the best characterized hallmarks of AD. Clinical evidence has shown the accumulation of Aβ plaque deposits in postmortem human brains (Allsop et al., 1983). *In vivo* studies with male senescence accelerated OXYS non-transgenic rats demonstrated an association between the increase in Aβ deposition and age in AD (Stefanova et al., 2015). The accumulation of amyloid deposits over time predisposes individuals to developing sporadic late-onset AD (Castellano et al., 2012). The amyloid cascade hypothesis asserts that aggregation of Aβ plaques leads to a cascade of events that includes the activation of microglia and astrocytes while propagating oxidative injury. However, clinical trials of drugs and/or biologics that target Aβ have failed, resulting in a redirection of efforts toward other targets (Kametani and Hasegawa, 2018; Hillen, 2019). While the Aβ pathway is regarded as an important marker of AD pathology, particularly at early stages of disease, there are ongoing challenges to the amyloid cascade hypothesis (Hardy and Selkoe, 2002; Levin et al., 2022).

In tandem to the increasing Aβ levels that occurs over the course of the lifetime, aberrant intracellular signaling results in hyperphosphrylation of tau, the critical neuronal structural protein. Hyperphosphorylated tau aggregates to form neurofibrillary tangles neurofibrillary tangles (NFT), ultimately disrupting synaptic transmission and inducing mechanisms of degeneration. The progression of disease is illustrated histopathologically by the pattern of NFT accumulation across the brain, originally characterized by Braak and Braak in AD stages I-VI (Braak and Braak, 1991b,^1995^). AD staging revealed the involvement of the limbic system and its autonomic implication in early onset of AD. The autonomic dysfunction worsens as the NFT stage increases (Allan et al., 2007; Tulba et al., 2020). Recent studies have demonstrated that by interacting with the α2a adrenergic receptor (AR), Aβ_42_ oligomers (Aβ_42o*lig*_.) can elicit the glycogen synthase kinase 3β (GSK3β)/tau cascade (Zhang F. et al., 2020).

In addition to the hallmark pathogenesis of AD marked by Aβ aggregation and tau phosphorylation, AD is also profoundly affected by neuroinflammatory processes. Thus, we are now also developing an understanding of the importance of astrocyte and microglial activation, as well as the release of cytokines and other inflammatory signaling molecules. The inflammation caused by both Aβ plaques and neurofibrillary tangles are exacerbated by microglial activation in the late stage of AD. Microglial activation releases cytokines, chemokines, and complement proteins. These detrimental effects of microglial activation are juxtaposed with their advantage of clearing aberrant protein aggregation in the acute stage of AD (Heneka et al., 2015). Thus, the proteinopathy-induced microglial activation along with inflammatory markers may exert an allostatic overload, propagating AD disease progression (Lee et al., 2015). Similarly, astrocytes are known to possess a dual nature of degrading Aβ plaques and contributing to cytokines and pro-inflammatory mediators (Wang et al., 2015; Frost and Li, 2017). Both microglia and astrocytes release nitric oxide (NO) which propagate tau hyperphosphorylation (Fakhoury, 2018). The oxidative stress, tauopathy, and amyloidogenic pathways synergistically impairs neuronal functioning and predisposes individuals to cognitive detriment (Tonnies and Trushina, 2017). Finally, individuals with AD are noted to have decreased Aβ clearance *via* vascular smooth muscle cells (VSMCs) due to the compromise of the blood brain barrier (BBB). The BBB is threatened by Aβ plaques, which causes microhemorrhages, hypoperfusion, and alteration of VSMCs (Tarasoff-Conway et al., 2015).

### Overview of stress integrative circuitry

#### Hypothalamic-pituitary-adrenal axis

**FIGURE 1:**
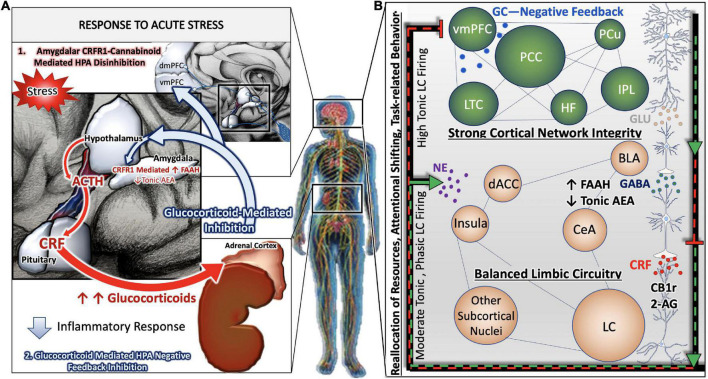
Stress-integrative circuitry intact and adaptive. Under normal physiological conditions, the HPA axis is regulated by negative feedback **(A)**. The activation of the HPA axis and subsequent synthesis and release of glucocorticoids from the adrenal glands feed back onto anatomical substrates such as the PVN and mPFC to terminate the stress response. Peripheral, acute CRF signaling and response by the CRF Receptor 1 on immune cells suppress the immune response. In parallel, negative feedback mechanisms of the central stress-integrative circuitry are intact and allow for adaptive responses to stressful stimuli. Under acute stress, FAAH activity is upregulated and AEA levels are decreased, enabling the activation of the HPA axis (Hill et al., 2009). The transient stress induced AEA levels in the amygdala plays an important role in balancing AEA signaling (Hill et al., 2010), and suggests that AEA tonically inhibits BLA activity in the absence of stressors (Hill et al., 2011a). Based on a series of neuroanatomical and biochemical experiments, we have put forth the idea that CB_1_r is strategically localized to presynaptic terminals of CRF-containing neurons that arise in the amygdala as a mechanism of regulating input from the amygdala to the LC. The peri-LC contains a majority of excitatory synapses, while the core LC possesses mostly inhibitory synapses. This supports the idea that amygdalar CRF afferents are modulated by the eCB system to adapt to emotional stimuli during the stress response **(B)**.

In this section, we highlight the topographic and internuclear regions of the nervous system involved in responding to cognitive and physical stressors. When exposed to a stressful stimulus, the paraventricular nucleus (PVN) of the hypothalamus releases adrenocorticotropic hormone (ACTH) onto the posterior pituitary. In effect, the pituitary gland secretes the neurohormone corticotropin releasing factor (CRF) into the hypophyseal portal system, allowing CRF to travel through the bloodstream to activate the adrenal glands in the periphery. Simultaneously, the central response to stress is activated when CRF is released from afferents of the PVN that project to the locus coeruleus (LC). The LC contains the most robust population of norepinephrine (NE) -producing neurons in the brain (Valentino and Van Bockstaele, 2008; O’Donnell et al., 2012). The activation or excitation of LC neurons results in the release of NE throughout nearly the entire neuraxis to coordinate a cognitive response that facilitates adaptive behavior and dynamic learning (Figure 1).

#### Coeruleo-cortico-amygdalar circuit

NE is responsible for maintaining a plethora of functions: agitation, aggression, arousal, attention, cognition, autonomic processes, and sleep-wake cycles (Berridge and Waterhouse, 2003; David and Malhotra, 2022). The LC is uniquely poised to coordinate endocrine, emotional, and cognitive aspects of the central response to stress. Not only is the LC linked with the HPA axis to receive and relay endocrine information, but it is also reciprocally connected with the amygdala and prefrontal cortex (PFC). It is thought that the central nucleus of the amygdala (CeA) conveys information regarding the emotional salience of a particular stimulus, while cortical projections are involved in facilitating adaptive behavior. The cognitive limb of the stress response revolves around the LC modulation of cortical and limbic regions (Figure 1B). LC-PFC bidirectional neuronal connections are vital in maintaining resiliency to various states of allostatic load by appropriately adapting flexible behavior to the stress response. According to Chandler and colleagues, the LC evokes set-shifting within the PFC that facilitates dynamic learning behavior (Chandler et al., 2019). This neurobehavioral adaptation is mediated by a subpopulation of LC neurons with DREADD-activated LC terminals within the PFC (Cope et al., 2019). Moreover, the LC-PFC is able to compartmentalize sensory and behavioral inputs while also appraising salient stimuli. This flexibility of top-down cortical processing dampens amygdalar activity at normal parameters. However, this top-down regulation is devastated under stress conditions (Arnsten, 2009). During chronic stress, the LC-NE discharge is heightened which leads to dysregulation of the PFC and recruitment of posterior cortical and subcortical regions. This is contrasted with acute stress where the executive function of the PFC can adjust behavior appropriately (Chandler, 2016; Figure 1).

Additionally, the PFC has a dual mechanism in terminating HPA axis *via* the short-loop and long-loop glucocorticoid negative feedback (Hill et al., 2011b). The short-loop involves regional glucocorticoid influences in the PVN, and the long-loop engages the corticolimbic circuitry in cross-communicating with the hypothalamus (Herman et al., 2003). Emphasis of the mPFC in the corticolimbic system is important to elucidate how cognitive and emotional salient stimuli are processed in a top-down regulation, thus influencing the HPA axis. Furthermore, the complexity of the mPFC is characterized by its diverse topographical nuclei. There exists a noteworthy differentiation between the dorsoventral axis of the mPFC, and its implication upon PVN regulated sympathoadrenal and neuroendocrine processes under acute emotional stress (Heidbreder and Groenewegen, 2003; Radley et al., 2006a). A retrograde tracing experiment in rats demonstrated increased Fos expression in the PVN with ventral mPFC lesions, suggesting a role in stress-induced PVN autonomic output (Radley et al., 2006a). The dorsal region, known as the prelimbic medial PFC (PL-mPFC) is responsible for mainly inhibiting the HPA axis, whereas the ventral region, known as the infralimbic mPFC (IL-mPFC) is known for activating the HPA axis (Radley et al., 2006a).

#### Acute and chronic stress responses involve coordinated immune responses

Under normal physiological conditions, the HPA axis is regulated by negative feedback (Figure 1A). The activation of the HPA axis and subsequent synthesis and release of glucocorticoids from the adrenal glands feed back onto anatomical substrates such as the PVN to terminate the stress response. Upon exposure to an acute stressor, the HPA axis and coeruleo-cortico-amygdalar circuitry are activated in parallel to facilitate an adaptive behavioral response and later inhibited by glucocorticoid signaling. Our laboratory has demonstrated that the LC is finely tuned by co-regulation of CRF, endogenous opioids and the eCB systems (Scavone et al., 2010; Cathel et al., 2014; Reyes et al., 2014; Wyrofsky et al., 2017, 2018). The level and mode of LC activity is dependent on the balance of neuromodulators that are active at a particular time.

**FIGURE 2:**
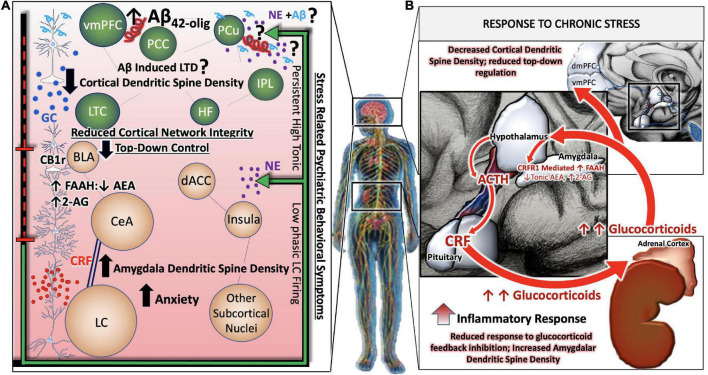
Allostatic overload renders stress-responsive circuits vulnerable to neuronal injury and degeneration. During states of chronic or pathological stress, the HPA axis, LC, and associated stress integrative circuitry no longer respond to negative feedback inhibition, resulting in aberrant, unregulated, activity of the entire system **(B)**. Chronic stress related psychiatric behavioral symptoms are the result of complex, between- and within- network adaptations. While large-scale cortical networks become less intrinsically connected, and the microcircuitry that comprise them retract in dendritic density and complexity, there is correspondingly less top-down regulation of limbic circuitry. GABA-ergic interneurons of the BLA are disinhibited by decreased cortical input, leading to greater activity in limbic regions such as the central nucleus of the amygdala (CeA). Further shifts away from cortical control and toward limbic control occur as CeA afferents release increasing amounts of CRF in high frequency onto locus coeruleus (LC) neurons, rendering them more susceptible to stress-related excitotoxicity. A predominance of CB_1_r are found on cholecystokin (CCK)-positive GABAergic interneurons in the BLA. The inhibition of the BLA *via* CCK-positive GABAergic interneurons may be able to restore homeostatic LC tone by inhibiting CeA projections to the LC. In effect, this would silence HPA hyperactivation and halt the propagation of BLA excitability in a state of allostatic overload **(A)**.

In contrast, during states of chronic or pathological stress, the HPA axis, LC, and associated stress integrative circuitry no longer respond to negative feedback inhibition, resulting in aberrant, unregulated, activity of the entire system (Figure 2B). Under conditions of chronic stress, the prolonged neurotransmission of CRF onto LC dendrites expressing CRF receptor 1(CRFR1) results in the cellular adaptation known as sensitization, in which LC neurons become more sensitive to CRF input. Our group and others have gathered biochemical and physiological evidence for heightened excitability of LC neurons in the presence of CRF (Valentino et al., 1998; Curtis et al., 2006; Reyes et al., 2008). Furthermore, the CRF afferents originating in the CeA project to the peri-LC, whereas afferents originating in the PVN project to the core LC (Valentino et al., 1996; Van Bockstaele et al., 1998, 2001; Reyes et al., 2005). This topographical innervation of the LC is relevant given that CRF afferent distribution can be mapped to its specific nuclei function. Thus, perturbation of CRF is vital in mediating the LC-NE circuitry especially within the context of integrating arousal, contextualizing stressful stimuli, and preserving cognitive function (Figure 2A).

**FIGURE 3:**
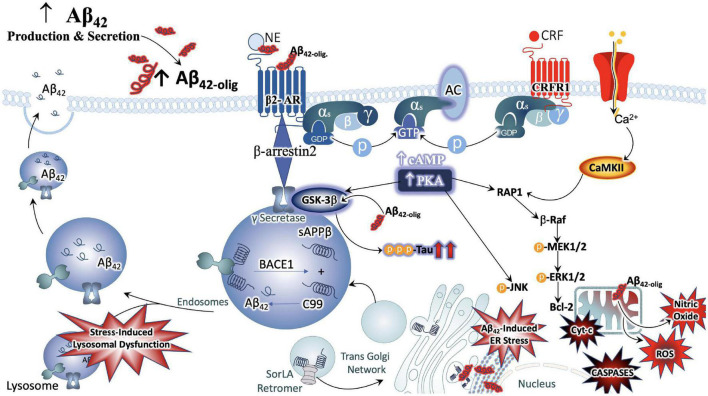
Convergence of chronic stress intracellular signaling with Aβ_42_ and hyperphosphorylated tau neuropathology. The noradrenergic and CRF systems are engaged during stress and may contribute to neuronal degeneration when sustained over long periods of time. Adrenergic and CRF receptors are known to shift APP processing toward the amyloidogenic pathway by influencing the activity of beta and gamma secretases, BACE1 and PSEN, respectively. Increased production and secretion of Ab42 results in increased production of the neurotoxic Ab42 oligomers, known to incite nitric oxide (NO), reactive oxygen species (ROS), lysosomal and endoplasmic reticulum (ER) stress. The beta-2 adrenergic receptor and CRF receptor 1 (CRFR1) are both G-protein coupled receptors that engage stimulatory alpha subunits. Activation of these receptors results in the activation of adenylyl cyclase (AC), increased production of cyclic adenosine monophosphate (c-AMP), and activation of the enzyme protein kinase A (PKA). PKA can activate GSK3b signaling, resulting in the phosphorylation of tau at Alzheimer’s relevant sites. PKA can also elicit intracellular calcium increases that result in the recruitment of the calcium modulatory kinase II (CaMKII). Sustained elevations in Ca2+ signaling, however, results in the activation of additional downstream cell signal cascades, exacerbating the production and release of NO, ROS, caspases, and cytochrome-c (Cyt-c) from mitochondria. Cumulatively, this results in decreased efficiency in clearing proteins from the cell, increased inflammation and signals that induce apoptosis, contributing to neuronal degeneration.

It is also noteworthy that the NE and CRF systems play a significant role in modulating inflammation (Feinstein et al., 2002; Heneka et al., 2002, 2015; Smith and Vale, 2006). CRFR1 is expressed in peripheral organs including the skin, reproductive organs, adrenal medulla, enteric nervous system, macrophages, mast cells (Cao et al., 2005), T- and B-cells of the inflammatory system as well as epithelial and endothelial cells (Lichlyter et al., 2022). During acute responses to stress, CRFR1 signaling results in protective cell signaling cascades, and the suppression of inflammation, allowing the body to adapt to stressful situations while maintaining cell viability (Elliott-Hunt et al., 2002; Facci et al., 2003; Radulovic et al., 2003). On the other hand, conditions of chronic stress or long-term CRF signaling results in the induction of alternate signaling pathways, including that of protein kinase c (PKC) (Kiang et al., 1994; Dermitzaki et al., 2005; Lesscher et al., 2008). Sustained levels of high Ca^2+^ recruits calmodulin and increases PKC signaling, resulting in the release of reactive oxygen species (ROS), initiating inflammatory cascades and releasing calpains (Hayashi et al., 1999; Vosler et al., 2008) that induce cell death (Lichlyter et al., 2022; Figure 3).

### Significance of allostatic overload for neuronal injury and degeneration

Allostatic Overload, or chronically elevated levels of glucocorticoids, combined with sustained CRFR1 signaling and coordinated inflammatory responses, is sufficient to increase neuronal vulnerability to excitotoxicity, induce metabolic changes in ATP production, induce endoplasmic reticulum stress (Zhang et al., 2012), decrease efficiency of mitochondrial function, as well as lower the seizure threshold (Rosen et al., 1994; Maguire and Salpekar, 2013) and promote vascular dysfunction of the blood BBB (Lichlyter et al., 2022). Moreover, several preclinical studies on models of chronic or repeated stress have demonstrated that stress signaling contributes significantly to aberrant tau hyperphosphorylation and production of the plaque-forming 42-amino acid Aβ proteins (Aβ_42_). One of the earliest reports supporting this line of inquiry demonstrated a shift in APP processing toward the amyloidogenic pathway in naïve, middle-aged rats exposed to chronic stress and excessive glucocorticoid production (Catania et al., 2009). Repeated stress has also been shown to increase levels of pre-pathological phosphorylated tau protein (Rissman et al., 2012). Mice that are genetically altered to globally overexpress CRF demonstrate brain atrophy at 3–6 months of age and show hippocampal learning deficits by 9 months (Campbell et al., 2015a,b). The significance of these observations are further highlighted by biochemical analyses that show increased levels of phosphorylated GSK-3β, increased phosphorylation of tau at AD-relevant AT-8 and PHF-1 sites (Campbell et al., 2015a,b). Other studies utilizing this model have reported decreased levels of neurotrophic support and a shift away from growth factors and toward cellular energy expenditure (Le et al., 2016).

A recent neuroanatomical study from our group utilized a genetic model of chronic stress to investigate the impact of chronic stress on endogenous rodent amyloid beta peptide distribution within noradrenergic circuitry (Ross et al., 2019). The mice were genetically modified to conditionally overexpress CRF (CRF-OE) specifically in the forebrain with doxycycline administration in the chow (Ross et al., 2019). We elected to administer doxycycline to the genetically modified animals for 2 weeks during adulthood, thus increasing CRF expression in the forebrain for 14 days prior to sacrifice and subsequent neuroanatomical and biochemical analysis. While overall Aβ_42_ levels did not change between the two treatment conditions, as measured by enzyme linked immunoassay (ELISA), our ultrastructural studies show a significant redistribution in the LC. The male and female CRF-OE mice showed a greater prevalence of Aβ_42_ peptides in the cell bodies of the LC, compared to their genetically modified littermates that did not receive doxycycline treatment. Moreover, CRF-OE mice of both sexes displayed abnormal morphology of the lysosomal compartments responsible for clearing Aβ_42_ peptides. The pericytes, specialized cells that surround the BBB and regulate brain blood flow, of CRF-OE female mice were swollen and contained lipid-laden vacuoles. CRF-OE mice of both sexes exhibited LC neurons that were positively labeled for Aβ_42_ surrounded by greater amounts of glial fibrillary acidic protein (GFAP) immunoreactive processes (Ross et al., 2019). Taken together, our findings suggest that cellular mechanisms of homeostasis are dysregulated in the LC of CRF-OE mice. Interestingly, various rodent models of AD show similar lysosomal (Choi et al., 2013) and BBB abnormalities (Aliev et al., 2002), and have been found to parallel the pathology evident in human AD brains (Aliev et al., 2002; Nixon et al., 2005). Additionally, within the LC of CRF-OE male and female mice, we observed a positive correlation between CRFR1 expression and tau phosphorylation at an AD-relevant site (Ser396) (Ross et al., 2019). Taken together, the studies summarized in this section demonstrate that stress signaling alone has significant consequences for indices of neuronal injury and degeneration independently of age and/or AD genotypes (Figure 3).

## Involvement of stress circuits in Alzheimer’s disease neuropathology

### Postmortem evidence

Braak and colleagues have suggested that the LC is amongst the earliest brain regions involved in AD neuropathology (Braak and Del Tredici, 2011b). Supported with evidence that pretangle material is present in the LC before any other brain region, and that soluble, non-toxic pretangle material can be found in the LC as early as before puberty or young adulthood (Braak and Del Tredici, 2011a,b). Taken together with the abundance of NFTs in AD in postmortem brain tissue, it is hypothesized that tau dysregulation originates in the LC, and gradually builds and spreads throughout the brain during the prodromal period decades before symptoms of behavioral and cognitive impairment (Braak et al., 2011). For a more detailed discussion of the previously underappreciated role of the LC in AD neuropathology, we direct the reader to previously published reviews by our group and others (see Gannon et al., 2015; Ross et al., 2015; Giorgi et al., 2020). Postmortem studies display a dramatic increase of neuronal loss in the LC, most notably the rostral region (Beardmore et al., 2021).

The depletion of LC noradrenergic neurons elicits a compensatory response that involves the upregulation of β-ARs in the hippocampus and prefrontal cortex, as evident in human AD postmortem brains (Kalaria et al., 1989; Sanders et al., 2006). Further evidence for compensation comes from biochemical analysis of the remaining LC neurons, which exhibit increased mRNA expression of NE biosynthetic enzymes, increased dendritic branching of the peri-LC, and altered projections to the hippocampus (Szot et al., 2006). Increased indices of NE function such as these, would suggest a change in noradrenergic transmission. Some existing studies indicate that NE levels are dynamic with low and unaltered concentrations in human AD brain regions (Adolfsson et al., 1979; Matthews et al., 2002; Gannon and Wang, 2019). Others have shown that plasma NE levels are decreased in humans with dementia type AD and displayed a positive correlation with Mini Mental State Examination (MMSE) score in human AD (Umegaki et al., 2000; Pillet et al., 2020). While the collective data on NE transmission levels are inconsistent, there is a prevailing theory that the compensatory changes in LC neurons maintain NE function, and may even over-compensate for a time, but ultimately results in neuronal fatigue and reductions in NE transmission. These alterations can have profound effects on the function and integrity of stress circuitry (Szot et al., 2006; Liu et al., 2018).

As a central hub for peripheral and central stress responses, circadian rhythms and global neuroendocrine function, the hypothalamus is a key region within the stress integrative circuitry that is profoundly influenced by AD pathology. Early studies established that Aβ plaque staging is present in the hypothalamus defined as stage 3 of AD in humans (Thal et al., 2002). More recent postmortem studies have identified hypothalamic plaques with distinct epitope variation from β-amyloid precursor proteins (β-APPs) (Baloyannis et al., 2015). NFT are also found within the hypothalamus, although the exact mechanism of tau pathology in the hypothalamus is unknown (McDuff and Sumi, 1985; Saper and German, 1987; Braak and Braak, 1991a; Ishii and Iadecola, 2015). One theory of tau pathology in the hypothalamus stems from anterograde LC-transneuronal projections (Iba et al., 2015). There is a profound reduction in noradrenergic neurons about 53% in the hypothalamus in postmortem AD studies (Mann et al., 1980). The decreased NE levels in the hypothalamus of dementia type AD brains compared to normal robust NE concentrations in postmortem brains (Farley and Hornykiewicz, 1977). Considering that the LC provides the majority of NE innervation to the hypothalamus (Farley and Hornykiewicz, 1977), it is possible that LC degeneration plays a role in dysregulation of the hypothalamus in AD. These observations support a model in which early LC dysfunction and degeneration contribute to HPA axis dysregulation, resulting in a chronic stress syndrome characterized by a reduction of glucocorticoid negative feedback and increased levels of circulating glucocorticoids. Although the neuropathological origin of hypothalamic dysfunction requires further investigation, its impact on the integrity of the HPA axis are reflected in the clinical finding that glucocorticoids are elevated in AD patients, and that high glucocorticoid levels are associated with increased risk of AD (Pedrazzoli et al., 2019).

### Preclinical evidence

#### The role of adrenergic receptors

NE serves a pivotal role in activating excitatory and inhibitory processes in the stress circuitry to maintain a homeostatic balance *via* α- and β- ARs (Hein, 2006). NE levels in the PFC play a major role in working memory and cognitive function. This is illustrated by the decline in spatial memory and reduction in NE concentration among rat models using an LC-selective neurotoxin (Sontag et al., 2008). In line with this, optimal NE levels in the PFC enhance working memory *via* presynaptic α1-AR. Notably, a reciprocal effect is seen when high stress-induced NE levels inhibit neuronal activity *via* postsynaptic α1-AR (Zhang et al., 2013). Cognitive impairment, expressed behaviorally as decreased open field exploration (Dvorkin et al., 2008), was shown in α1-AR subtype-b knockout mice exposed to stressful stimuli (Knauber and Muller, 2000). Dysregulation of the PFC cognition is improved with Betaxolol, a β1-AR antagonist, infused into the PFC of rats and administered systemically to monkeys (Ramos et al., 2005).

A complex relationship exists between AD neuropathological markers and adrenergic receptors in the PFC. Early studies identified α2-AR as contributors to age-related cognitive decline in the PFC (Arnsten and Goldman-Rakic, 1985). Moreover, it has been demonstrated that chronic treatment with the α-2AR antagonist fluparoxan, prevented age-related deficits in spatial working memory in APP/PS1 transgenic mice (Scullion et al., 2011). Mechanistically, α2aAR can regulate APP processing and Aβ production through an interaction with endocytic and secretory pathways (Chen et al., 2014). It has also been demonstrated that by interacting with α_2a_AR, Aβ oligomers can elicit the GSK3b/tau cascade (Zhang F. et al., 2020). In contrast, APP23 transgenic mice administered prazosin, an α1AR antagonist, sustained memory deficits while also displaying decreased Aβ concentration *via* APP processing in ELISA and Western blot analyses (Katsouri et al., 2013).

β2-AR activation by clenbuterol, a β2-AR selective agonist, infused into the PFC of rats and injected intraperitoneally in amyloid precursor protein/presenilin 1 (APP/PS1) mice improved PFC functioning in rats as well as hippocampal learning and memory (Ramos et al., 2008; Chai et al., 2016). According to Wu and colleagues, 6 month old female amyloid precursor protein/presenilin 1 double transgenic (APPswe/PS1dE9) mice chronically given a β2-AR selective antagonist (ICI 118,551, 1 mg/kg daily i.p. for 2 months) revealed a poor spatial learning compared to wild type mice in the Morris water maze and increased Aβ levels in the hippocampus *via* downregulation of α-secretase activity (Wu et al., 2017). On the contrary, there are earlier reports of elevated Aβ levels in APPswe/PS1dE9 derived cells exposed to chronic β2-AR agonist isoproterenol, *via* a cAMP-independent, endocytosis-dependent enhancement of γ-secretase activity (Ni et al., 2006). *In vivo* experiments reported in this study indicate that male and female APPswe/PS1dE9 mice (up to 5 months old) exposed to NE (icv 2 μg daily) or selective β2-AR agonist clenbuterol (2 mg/kg per day *via* cannulae) for 30 days exhibited increased cerebral Aβ plaques. Chronic administration of the β2-AR selective antagonist ICI 118,551 (1 mg/kg daily) ameliorated plaque pathology in APPswe/PS1dE9 mice (Ni et al., 2006). A third study demonstrates that hippocampal injections of β_2_-AR-selective agonist clenbuterol enhanced the production of acute stress-induced Aβ peptide production while the β_2_-AR-selective antagonist (ICI 118,551) reduced Aβ peptide production (Yu et al., 2011). In line with these results, the absence of β2-AR in APP/PS1 mice attenuated the pathogenesis of AD in the PFC (Wang et al., 2013). Overall, these studies have shown mixed results of β2-AR activation and inhibition on memory and synaptic plasticity and dendritic spine density in AD. These data obscure the therapeutic potential of directly targeting adrenergic receptors in AD, especially at later stages of disease. Nonetheless, the LC-NE system in AD neuropathology and the involvement of adrenergic receptors should be further investigated.

#### Contribution of locus coeruleus-norepinephrine-hypothalamic-pitu itary-adrenal axis interactions: The role of glucocorticoids and corticotropin releasing factor

The LC-NE circuitry exerts excitatory effects upon the HPA axis resulting in a downstream cascade of neurohormones and transmitters. Under chronic stress, there is increased noradrenergic tone and enhancement of the HPA axis activation. In contrast to the studies described in section “Significance of allostatic overload for neuronal injury and degeneration” this section will focus on studies that have combined AD models with behavioral paradigms of stress or genetic alterations that increase stress signaling. Interestingly, it has been reported that rodent models of AD have an intrinsic increase in central stress system drive as well as decreased negative feedback inhibition (Elgh et al., 2006; Hebda-Bauer et al., 2013). One report indicated that APP/hAβ/PS1 mice had an intrinsic increase in CRF expression in the PVN, hippocampus and amygdala (Guo et al., 2012). Thus, LC-NE-HPA dysregulation may be uniquely involved in activating the central drive and feeds into aberrant disease processes.

The sustained amplification of glucocorticoids and CRF are closely associated with amyloidogenic pathways and tauopathies (Catania et al., 2009). One study found a direct effect of glucocorticoids on the Aβ burden and tau pathology (Swinny and Valentino, 2006). It was also reported that the overactivation of the HPA axis can eventually increase the risk for developing senile plaques (Lesuis et al., 2018). Moreover, it has been demonstrated that increased glucocorticoid levels induce Aβ deposition and tauopathy within the hypothalamus in a rodent model of AD (Green et al., 2006). Researchers suggest that proteinopathy may play a role in positive feedback to further propagate glucocorticoid levels (Green et al., 2006). This sheds light on “the chicken or the egg” phenomenon between the concomitant relationship between elevated Aβ and tau deposition. According to De Souza and colleagues, there are low levels of CRF immunoreactivity in the cortex of postmortem human AD brains. This study found an inverse relationship between decreased CRF immunoreactivity and upregulated CRF receptors in AD (De Souza et al., 1986). Furthermore, the overexpression of CRF in AβPP+/CRF+/tTA+ transgenic mice revealed increased Aβ burden in the cortex and dendritic loss (Dong et al., 2012). This sets the precedence of CRF receptors type 1 in modulating the pathophysiology of AD.

Several preclinical studies have demonstrated that stress or increased CRF receptor 1 (CRFR1) signaling during stress contributes significantly to aberrant tau hyperphosphorylation and production of Aβ_42_, accelerating AD neuropathology (Kang et al., 2007; Dong et al., 2012). Studies that utilized the Tg2576 AD rodent model combined with isolation stress demonstrated that increased plasma corticosterone and glucocorticoid receptor levels were positively correlated with increased Aβ_42_ plaque deposition (Dong et al., 2004, 2008), as well as decreased hippocampal neurogenesis that corresponded with impaired contextual memory (Dong et al., 2004). Cognitive decline in this model could be rescued with the glucocorticoid antagonist RU486 (Lante et al., 2015). Overexpression of CRF in AβPP transgenic mice showed increased Aβ_42_ burden in the cortex as well as dendritic loss (Dong et al., 2012). In contrast, transgenic PSAPP mice crossed with mice null for CRFR1 (CRFR1-KO-PSAPP) had lower levels of Aβ_40_ and Aβ_42_, and decreased levels of AβPP C-terminal fragments compared to PSAPP mice. The concurrent finding that Insulin Degrading Enzyme, a metabolizing enzyme of Aβ peptides, was also decreased in this model led investigators to suspect that the CRFR1-dependent mechanism is based on production of Aβ peptides rather than clearance (Rissman et al., 2012). Further studies by this group examined the effects of CRFR1 activation, inhibition, or ablation on the phosphorylation of tau (tau-p), indicated that acute stress induced tau-p was transient and potentially important for neuroplastic adaptations to stress (Rissman et al., 2007). In contrast, tau-p induced by repeated stress or overexpression of CRF was present for extended periods after stress exposure, had a definable structure and was localized to detergent soluble cellular fractions (Rissman et al., 2012; Campbell et al., 2015a). Thus, tau-p induced by chronic stress *via* CRFR1 forms pre-pathological structures that may result in NFT formation over long periods of time (Figure 3).

Because CRF is a critical linking factor of LC-NE and HPA axis function, these data suggest that pharmacological targeting of CRF-R1 and other CRF-related targets may have therapeutic potential as an early stage intervention to halt the progression of AD pathology (Vandael and Gounko, 2019). Taken together, these studies indicate a bidirectional relationship in which stress circuitry is intrinsically dysregulated in AD rodent models and AD neuropathology is exacerbated by exposure to behavioral stress and/or augmented stress signaling (Figure 3 and Table 1).

**TABLE 1:** Influence of stress/stress signaling.

	AD neuropathology	Neuroinflammation	Other
**AD transgenic rodent models**	Tg2576 + Isolation stress: ↑ plasma corticosterone ↑ GC receptor levels ↑ Ab plaque deposition (Dong et al., 2008). ↑Ab production ↓neurogenesis (hippocampus) (Dong et al., 2004). CRF overexpression AβPP + /CRF + /tTA + transgenic mice ↑ Aβ burden in the cortex and dendritic loss (Dong et al., 2012). CRFR1-KO-PSAPP Mice vs. PSAPP ↓ Aβ40, Aβ42 ↓ AβPP CTFs ↓ Insulin degrading enzyme (IDE): mechanism may involve Aβ production rather than clearance (Campbell et al., 2015a,b).	APP/hAβ/PS1 mice Intrinsic ↑ CRF PVN, hippocampus, amygdala (Guo et al., 2012). 11-month old 5x FAD Mice Intrinsic ↓ CB1r ↓MAGL ↑AGLL ↑ CB2r Hippocampus (Medina-Vera et al., 2020).	Tg2576 + Isolation stress: Impaired contextual memory (Dong et al., 2004). Tg2576 cognitive decline rescued with GC receptor antagonist RU486 (Lante et al., 2015).
**Relevant exposures/modifications** **in non-AD tg rodents/cells**	Chronic stress and excessive GC production: (middle aged rats) -APP processing shifts toward the amyloidogenic pathway (Catania et al., 2009). Repeated stress: ↑tPre-pathological Tau-p (Rissman et al., 2012). CRF-overexpressing mice: Brain atrophy at 3–6 months of age ↑ p- (GSK-3B) ↑ MAPK ↑ p38 ↑ ERK1/2. ↑ JNK-mediated tau-p at sites AT8, PHF-1 ↑tau aggregates (Campbell et al., 2015a,b). ↓CREB activation ↓ BDNF transport ↑BDNF transport velocity and distance traveled (Le et al., 2016).	Repeated stress: In mPFC, hippocampus: ↑ Dendritic atrophy ↓ glial counts ↓ proliferation (McEwen and Magarinos, 2001; Radley et al., 2004; Banasr and Duman, 2007; Banasr et al., 2007; Radley et al., 2008). Chronic unpredictable stress (CUS) ↓neurotrophins ↓ hippocampal neurogenesis ↓ dendritic arborization ↑arborization ↑arborization (Farooq et al., 2012; Campos et al., 2013b,^2017^)	CRF-overexpressing mice: -Brain atrophy (3–6 months) -Hippocampal dependent learning deficits (9 months) (Campbell et al., 2015a,b). Chronic unpredictable stress (CUS): **↑**anxiety and depression-like behaviors ↓cognitive performance (Farooq et al., 2012; Campos et al., 2013b,^2017^)

## The endocannabinoid system

### Broad overview of the endocannabinoid system

The endocannabinoid system (eCB) is a complex network involved in maintaining homeostasis. It is composed of endogenous ligands, receptors, enzymes, and signaling molecules. The widely known endogenous endocannabinoids are arachidonoyl ethanolamide (AEA) and 2-arachidonoylglycerol (2-AG). The synthesis of AEA is carried out *via N*-acyltransferase (NAT) and *N*-acylphosphatidyl-ethanolamine-specific phospholipase D (NAPE-PLD); whereas 2-AG is facilitated *via* phospholipases C (PLC) and diacylglycerol lipases (DAGLs). AEA and 2-AG are inactivated by enzymes fatty acid amide hydrolase (FAAH) and monoacylglycerol lipase (MAGL), respectively. Ultimately, endocannabinoid signaling is primarily relayed through endocannabinoid receptors that are composed of G protein coupled receptors (GPCRs). Cannabinoid 1 receptors (CB_1_r) are psychoactive receptors typically found in the brain with some present in the periphery. CB_1_r are primarily associated with neurons and microglia and are involved in modulating memory, cognition, emotion, and motor coordination. CB_2_r are also located in the brain, particularly under conditions of disease, but are better known as the endocannabinoid receptor of peripheral tissue. AEA is a partial agonist with a stronger affinity at cannabinoid 1 receptor (CB_1_r) than cannabinoid 2 receptor (CB_2_r), and 2-AG is a full agonist at both CB_1_r and CB_2_r (Mackie et al., 1993; Savinainen et al., 2001; Howlett et al., 2002). 2-AG is present an overall greater proportion than AEA throughout the whole brain (Zou and Kumar, 2018). Specifically, there exists a higher quantification of 2-AG levels in the PFC, hippocampus, limbic structures, and hypothalamus compared to AEA concentrations in these regions (Buczynski and Parsons, 2010).

#### Pharmacological tools used in cannabinoid research

A number of pharmacological tools exist that have facilitated research on the endogenous cannabinoid system as well as in the investigation of exogenous cannabis effects. The synthetic cannabinoid WIN-55,212-2 is a potent non-selective agonist that acts at both CB1r and CB2r. CBD is a naturally occurring non-selective weak agonist at both CB1 and CB2 receptors. However, recent studies indicate that CBD action is better understood as a negative allosteric modulator of CB1 that can antagonize the effects of THC and/or AEA (Laprairie et al., 2015). Moreover, CBD has been shown to inhibit FAAH activity (De Petrocellis et al., 2011). JWH-133 is a synthetic CB2r selective agonist, while Arachidonyl-2′-chloroethylamide (ACEA) is a synthetic CB1r selective agonist. In pre-clinical and clinical studies an indirect approach to increase eCBs has predominated due to the unwanted biphasic and psychoactive effects of targeting CB1r directly with (high potency) THC. We direct the reader to an excellent recent review on the pre-clinically and clinically observed effects of indirectly acting cannabinoid drugs (Paredes-Ruiz et al., 2021). In the remainder of this section, we will briefly discuss the most recent findings on the most widely used agents.

Compounds that inhibit the 2-AG degrading enzyme, MAGL, effectively increase levels of 2-AG while FAAH-inhibitors increase endogenous levels of AEA. A commonly used FAAH-inhibitor, URB597, has been shown to improve inflammatory and pain conditions in several models of neurodegeneration, as well as a wide variety of central nervous system disorders that include stress, migrane and depression (Paredes-Ruiz et al., 2021). However, URB597 has also been reported to have no effect on indices of inflammation and cognitive deficit as measured by the Morris water maze test in 5xFAD mice (Vazquez et al., 2015). Interestingly, the same study demonstrated significant beneficial effects on 5xFAD inflammation and cognition by genetically ablating FAAH, supporting the idea that pharmaceutical and genetic manipulation of FAAH have differential effects (Vazquez et al., 2015). Despite inconsistencies in the literature, and one clinical trial with an off-target drug effect related devastating outcome (Kerbrat et al., 2016), FAAH inhibitors are thought to hold the most therapeutic potential and are currently in development by several pharmaceutical companies. Currently in the clinic, PF-04457845 has demonstrated efficacy in reducing symptoms of Cannabis withdrawal (D’Souza et al., 2019), and in reducing stress responses by increasing AEA (Mayo et al., 2020), and has significant promise for the treatment of post-traumatic stress disorder (PTSD) (Mayo et al., 2022). While FAAH inhibitors have potentially broad ranging applications, MAGL inhibitors are thought to be more relevant for inflammatory conditions. One of the most studied MAGL inhibitors, JZL184, has been shown to decrease the reactivity of glial cells while concurrently reducing the production of pro-inflammatory mediators in several models in neurodegenerative disease, though none immediately pertinent to AD (Paredes-Ruiz et al., 2021).

A variety of endocannabinoid reuptake inhibitors (eCBRIs) also exist that prevent the releasing neuron from reabsorbing eCBs by preventing membrane transport, thereby increasing levels of eCBs in the synaptic cleft and prolonging endogenous cannabinoid action (Chicca et al., 2017). One of the most promising eCBRIs, N-(3,4-dimethoxyphenyl) ethyl amide (WOBE437), irreversibly blocks membrane transport of AEA and 2-AG, increasing extracellular levels of both endogenous cannabinoids without effecting any other N-acylethanolamines (NAEs). *In vivo*, WOBE437 elicits cannabinoid receptor dependent analgesia as well as reductions in inflammation resulting from LPS challenge. WOBE437 also attenuated anxiety behaviors when injected directly into the BLA (Chicca et al., 2017). WOBE437 was found to be orally bioavailable in the brain and exerted anti-inflammatory effects *via* CB1r (Rock et al., 2015), CB2r (Reynoso-Moreno et al., 2017) and PPARγ receptors (Rockwell et al., 2006). Moreover, WOBE437 was found to reduce disease progression in a model of multiple sclerosis (Reynoso-Moreno et al., 2021). However, another group working with WOBE437 recently reported off-target interactions as well as the surprising finding that WOBE437 increased AEA uptake by interacting with NAEs (Gagestein et al., 2022). The source of these inconsistencies is not clear.

#### Unique features of cannabinoid signaling and neuronal excitability

Cannabinoid modulation of neuronal excitability and synaptic activity is a functional component of the stress-buffering system that is heavily reliant on transcellular and intracellular signaling. Depolarization induced suppression of inhibition (DSI) of GABAergic terminals and depolarization induced suppression of excitation (DSE) of glutamatergic terminals are key mechanisms of cannabinoid mediated synaptic plasticity (Diana and Marty, 2004) that have significant functional implications for stress circuitry. For example, 2-AG mediated DSE of a glutamatergic hippocampal projection to the BLA in a model of traumatic stress was demonstrated using a combined approach of optogenetics and *ex vivo* electrophysiology (Bluett et al., 2017). Depolarization and consequent rise in intracellular calcium levels results in the post-synaptic release of 2-AG, which retrogradely travels across the synaptic cleft to activate CB1R on presynaptic terminals, ultimately inhibiting the release of neurotransmitter (glutamate in the case of DSE and GABA in the case of DSI) (Diana and Marty, 2004). Thus, cannabinoids have the unique ability to regulate pre-synaptic neurotransmitter release in an activity-dependent manner. On the other hand, conventional synaptic transmission of cannabinoids is thought to occur primarily *via* presynaptic release of AEA and postsynaptic receptor binding at projection sites (Castillo et al., 2012; Zou and Kumar, 2018). In both scenarios, pre- or post-synaptic CB1R activation signal through the Gα_*i*_ subunit of the heterotrimeric G-proteins, effectively decreasing adenylyl cyclase production and cAMP levels.

### Cannabinoid-mediated effects on stress circuitry

#### Neuroanatomical perspective: The endocannabinoid system is anatomically positioned to modulate stress circuitry

##### Coeruleo-cortical circuit

Findings from our research group and others provide compelling evidence that the eCB system may serve to modulate the LC-NE system to maintain an optimal level of activity. It has been established by many research groups that low tonic-low phasic firing of LC-NE neurons results in cognitive disengagement while high tonic-low phasic firing results in hyperarousal and difficulty sustaining attention on a given task. The optimal LC discharge rate for focused attention is in between the two, where phasic and coupled firing occurs (Berridge and Waterhouse, 2003).

The eCB system may regulate discharge rate locally within or near LC neurons. Evidence for tonic eCB production in the LC exists, as sole application of a CB1r antagonist is capable of decreasing LC-NE activity (Muntoni et al., 2006; Carvalho and Van Bockstaele, 2012). When LC-NE neurons are excited *via* bath application of potassium chloride (KCl), CB1r agonist pre-treatment can attenuate the KCl-induced increases in LC-NE firing (Mendiguren and Pineda, 2007), suggesting that the eCB system might function to prevent over-activation of LC-NE neurons. We have also demonstrated that, in the LC, CB1r are localized to glutamatergic, GABAergic and CRF presynaptic axon terminals that synapse with LC neurons (Wyrofsky et al., 2017). Thus, LC neurons may be substrates of DSI and DSE cannabinoid mechanisms of action. In addition, we provided ultrastructural evidence that CB1r is localized post-synaptically in somatodendritic processes of LC cells (Scavone et al., 2010). The presence of CB1r on LC-NE neurons is functional, as indicated by our electrophysiological studies, as well as that of other research groups, showing that CB1r agonists and FAAH inhibitors increase the basal firing rate of LC-NE cells, c-Fos expression of LC neurons, and NE efflux in the mPFC (Gobbi et al., 2005; Oropeza et al., 2005; Mendiguren and Pineda, 2006; Muntoni et al., 2006; Page et al., 2008).

Cannabinoid-mediated regulation of noradrenergic cells in the PFC is complex and multifaceted. Anatomically, CB1r is positioned at mPFC axon terminals of noradrenergic neurons and abundantly located in the infralimbic mPFC (Oropeza et al., 2007). Moreover, our lab identified a predominant population of excitatory synapses with both CB_1_r and α2AR localized at axon terminals in this region (Reyes et al., 2017). Thus, noradrenergic terminals of the mPFC contain two substrates for synaptic regulation that are informed by levels of NE (*via* α2AR autoregulation) and post-synaptic activity (*via* CB_1_r). To this point, we have reported that 2-AG is capable of retrogradely binding CB_1_r on presynaptic noradrenergic neurons to affect NE efflux. CB1r activation or inhibition could facilitate or inhibit NE efflux upon administration of a CB_1_r agonist and/or CB_1_r antagonist in an activity dependent manner (Oropeza et al., 2005; Page et al., 2008). Further, we have defined the anatomical substrates for this complex interaction between cortical noradrenergic neurons and neurons that produce 2-AG in the cortex *via* triple labeling of CB_1_r + DβH + DAGL-α (Reyes et al., 2015).

##### The role of the amygdala and the hypothalamic-pitu itary-adrenal axis

Cannabinoids are key modulators of the central stress response. As previously mentioned, CB_1_r can modulate the activation of the LC-NE system *via* CRF signaling in the HPA axis. Interestingly, glucocorticoid release within the PVN heightens CB_1_r signaling to attenuate CRF-secreting neurons (Evanson et al., 2010). Our lab has characterized CRF-projecting neurons from the central nucleus of the amygdala (CeA) colocalized with CB_1_r and either excitatory or inhibitory synapses within the rostrolateral peri-LC using immunofluorescence and electron microscopy (Wyrofsky et al., 2017). The rostrolateral peri-LC is relevant to AD neuropathology because AD postmortem studies indicate a majority of LC neuronal loss occur in this region (Beardmore et al., 2021). Based on a series of neuroanatomical and biochemical experiments, we have put forth the idea that CB_1_r is strategically localized to presynaptic terminals of CRF-containing neurons that arise in the amygdala as a mechanism of regulating input from the amygdala to the LC. As reported by Wyrofsky and colleagues, the peri-LC contains a majority of excitatory synapses, while the core LC possesses mostly inhibitory synapses. This supports the idea that amygdalar CRF afferents are modulated by the eCB system to adapt to emotional stimuli during the stress response (Figure 1B).

While a central focus of research on the effect of conditioned emotive stimuli within the limbic system has been dedicated to the CeA, it may also be important to consider the basolateral amygdala (BLA). The LC-BLA circuitry is involved in retrieval, consolidation, and extinction of memory. These functions can become disrupted by CeA-mediated regulation of the BLA during chronic stress (Roozendaal et al., 2002; Hollis et al., 2016). Bedse and colleagues report a predominance of CB_1_r on cholecystokin (CCK)-positive GABAergic interneurons in the BLA. The inhibition of the BLA *via* CCK-positive GABAergic interneurons may be able to restore homeostatic LC tone by inhibiting CeA projections to the LC. In effect, this would silence HPA hyperactivation and halt the propagation of BLA excitability in a state of allostatic overload (Bedse et al., 2015; Figure 2A).

Multiple substrates of the eCB system are effective inhibitors of stress related activity in the amygdala. Pharmacological studies demonstrated the suppression of corticosterone induced HPA axis activity *via* FAAH inhibitors in the BLA. Under acute stress, FAAH activity is upregulated and AEA levels are decreased, enabling the activation of the HPA axis (Hill et al., 2009). The transient stress induced AEA levels in the amygdala plays an important role in balancing AEA signaling (Hill et al., 2010), and suggests that AEA tonically inhibits BLA activity in the absence of stressors (Hill et al., 2011b). Additionally, CB_1_r signaling in the BLA helps to attenuate the HPA axis. CB_1_r agonist HU-210 administered in the BLA dramatically lowered corticosterone levels in rats (Hill and Gorzalka, 2006; Hill and McEwen, 2009; McLaughlin et al., 2009). It is noteworthy that the endogenous endocannabinoids effect within different brain regions slightly differs in mice models (Patel et al., 2005; Rademacher et al., 2008). There seems to be distinct species differences concerning endocannabinoid representation between mice and rat models. The differences may be attributed to the variability in cannabinoid receptor isoform expression and splicing and disparities in conserved cannabinoid receptor genes (Griffin et al., 2000; Zhang et al., 2015). In addition, some scientists believe that the disparities could be due to the unnoticeable, transient endocannabinoid levels which may oscillate during the stress restraint intervals (Gorzalka et al., 2008).

### Cannabinoids counteract the deleterious effects of stress in Alzheimer’s disease

#### Leveraging endocannabinoid neuroanatomy to protect stress-responsive circuits

The synchronous firing of neurons is critical for learning and memory processes (Jutras and Buffalo, 2010), and is significantly disturbed in AD models with increased production of Aβ and in humans with AD (Palop and Mucke, 2010a,b). These abnormal patterns of neuronal activity are observed as epileptiform activity in EEG recordings (Palop et al., 2007). Further investigation of individual neurons with high levels of Aβ and within abnormally firing circuits are either hyperactive or hypoactive relative to the intermediate activity of healthy neuronal controls (Busche et al., 2008). It has also been demonstrated that chronic neuronal hyperactivity is a driving force in Aβ accumulation (Yamamoto et al., 2015). Our research group has put forth the hypothesis that the aberrant activation of LC neurons during chronic stress in mid-late stages of life may render the brain more vulnerable to Aβ accumulation and senile plaque deposition, with consequent functional deficits in neuronal synchronization (Figure 5). As previously discussed, the eCB system is anatomically poised to modulate local activity of LC neurons by CB1r-mediated control of presynaptic glutamate, GABA or CRF release. Importantly, we also have described activity-dependent CB1r-mediated control of NE release from terminals in the widespread projection areas of the LC, including the PFC. To this point, it has been demonstrated that bilateral injection of selective CB1r agonist ACEA with Aβ prevented Aβ induced changes in evoked neuronal activity. Aβ administration alone reduced neuronal activity, an effect that was restored by cortical ACEA injection. Moreover, cortical ACEA injection rescued memory to baseline after the bilateral administration of Aβ peptides into the PFC of adult rats (Haghani et al., 2012).

**FIGURE 4:**
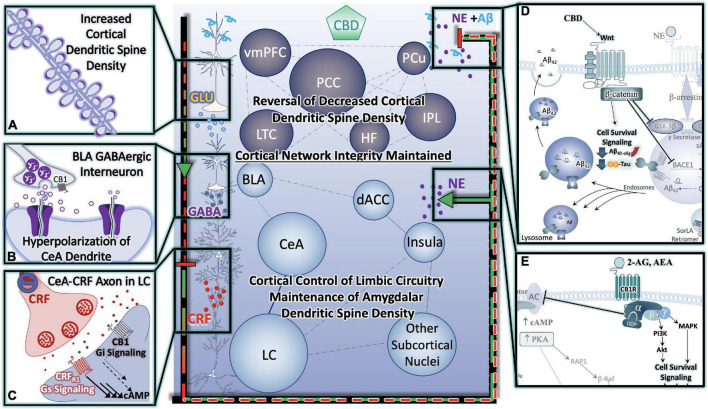
Cannabinoids Prevent Hyper-Activation of Stress-Integrative Circuitry, Protecting Cortical Network Integrity, Reducing Cellular Stress, Inflammation, Reduce Behavioral Symptoms and Improve Cognitive Function. **(A)** Cannabinoid-induced reversal of stress-induced dendritic retraction in cortical areas. **(B)** Cannabinoid-mediated engagement/activation of GABAergic interneurons of the BLA- reduces central drive to the CeA. **(C)** Cannabinoid-mediated reduction in central drive of the CeA results in downstream reductions in excitatory input to LC neurons, protecting them from consequences of hyperactivation. **(D)** Cannabinoids (including CBD) protect neurons from **(D)** Alzheimer’s Neuropathology Neuropathology, and **(E)** excitotoxicity and/or hyperactivity *via* Gαi signaling **(D,E)** are depicted in greater detail in Figure 6.

**FIGURE 5:**
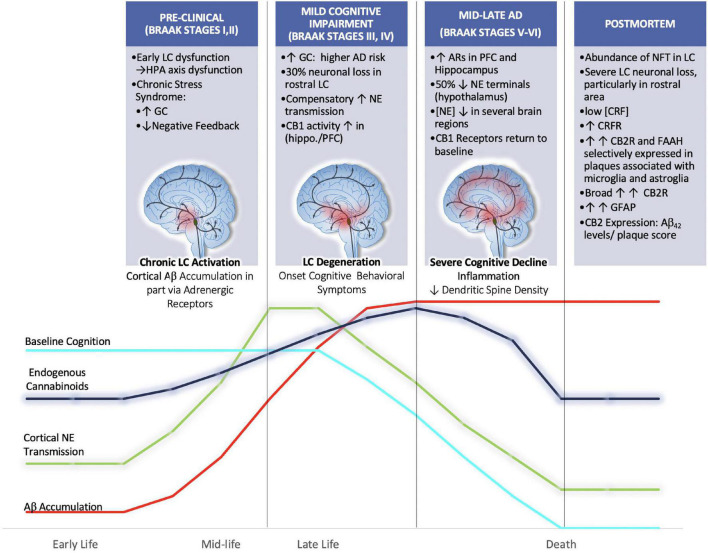
We hypothesize that aberrant activation of LC neurons during chronic stress in mid-late stages of life may render the brain more vulnerable to Aβ accumulation and senile plaque deposition. Further we hypothesize that endogenous cannabinoid compensation occurs in response to LC-NE system dysregulation preceding and after the onset of LC neurodegeneration. Clinical postmortem data show that between the prodromal (preclinical; Braak Stages I,II) and mild cognitive impairment (Braak stages III-IV) there is a 30% decrease in rostral LC volume (Theofilas et al., 2017). Remaining LC neurons compensate for neuronal loss by increasing NE production and transmission (Szot et al., 2006). Human AD brains in Braak Stages III, IV show increased CB1 activity in hippocampal and inner frontal cortical layers (Manuel et al., 2014). By the time disease progression reaches Braak stages V, VI there is human postmortem evidence for the upregulation of ARs in the PFC and hippocampus, thus suggesting that LC degeneration is causing decreases in NE transmission at this point (Kalaria et al., 1989). The loss of NE tone is supported by studies that have reported decreased concentrations of NE in the hypothalamus, cingulate, putamen and raphe nuclei (Mann et al., 1980; Arai et al., 1984). Further, during Braak stages V, VI, it has been shown that CB1r levels are decreased from their elevated state, returning to baseline levels (Manuel et al., 2014).

**FIGURE 6:**
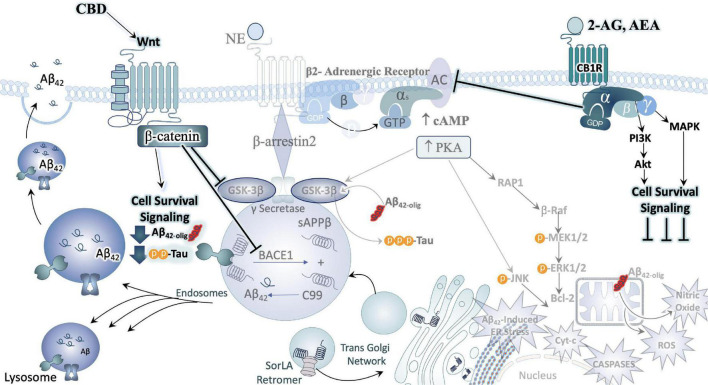
Cannabinoids counteract AD neuropathology exacerbated by stress. In direct opposition to (chronic) stress-responsive intracellular signaling cascades such as those downstream of the adrenergic receptors (ARs) or CRFR1, Cannabinoids inhibit cell signaling cascades that promote neuronal cell death. A major effector engaged by cannabidiol (CBD) exposure is the Wnt/β-catenin pathway. By binding its cognate receptor and co-receptor, Wnt increases the level of stable intracellular β-catenin proteins. β-catenin then goes on to inhibit GSK3β, the enzyme responsible for the upregulation of tau-phosphorylation, inhibit the beta-secretase (BACE-1), and promote the neuroprotective alpha-secretase cleavage of APP (not depicted here). Moreover, activation of CB1 receptors (CB1R) can counteract excessive stimulatory G-alpha subunit signaling by inhibiting adenylyl cyclase (AC) activity. By engaging the mitogen activated protein kinase (MAPK) and Akt pathways, CB1R signaling instead promotes the production and release of protective, cell survival factors.

Cognitive deficits observed in stress-related disorders including AD, arise from PFC dysfunction. As a crucial component of the cortico-limbic neurocircuitry, the mPFC has extensive downstream projections to regions as diverse as the amygdala and the brainstem (Sesack et al., 1989), providing a mechanism of executive top-down regulation of autonomic and neuroendocrine balance (Thayer and Brosschot, 2005), with influences on parasympathetic (Thayer and Sternberg, 2006) and HPA activity (Diorio et al., 1993). Of particular importance, are the excitatory projections from the mPFC that synapse onto gamma-aminobutyric acid (GABA)ergic interneurons of the basolateral amygdala (BLA), which suppress the output of central nucleus of the amygdala (CeA) neurons. This has significant implications, because in the absence of top-down modulation from the mPFC, hyperactivation of the CeA may result in increased LC neuronal activity and NE secretion, creating a vicious cycle of enhanced emotional and cognitive limbic excitability and reduced cortical control of those circuits (Ross et al., 2018). During the stress response, there is an upregulation of endocannabinoid signaling associated with the presence of glucocorticoids in both *in vitro* and *in vivo* studies (Di et al., 2003; Coddington et al., 2007; Hill and McEwen, 2009; Hill et al., 2010). Electron microscopy studies revealed a predominant presence of CB_1_r localized on GABAergic terminals adjacent to calcium/calmodulin-dependent kinase II-immunolabeled pyramidal neurons in the prelimbic mPFC (Hill et al., 2011b). Thus, the activation of CB_1_r are strategic in suppressing GABA secretion at the terminus while disinhibiting principal projection neurons in the prelimbic mPFC (Chiu et al., 2010). This ultimately leads to an attenuation of the HPA axis (Hill et al., 2011b; Figure 4).

It is possible, though somewhat speculative, that endogenous cannabinoid compensation occurs in response to LC-NE system dysregulation preceding and after the onset of LC neurodegeneration (Figure 5). The available literature that supports this idea primarily comes from AD postmortem brain tissue analyses that have stratified samples based on Braak staging. Using stereotaxic automated microscopy to assess postmortem AD brain tissue, it was discovered that between the prodromal (preclinical; Braak Stages I, II) and mild cognitive impairment (Braak stages III-IV) there is a 30% decrease in rostral LC volume (Theofilas et al., 2017). At this time, it has been demonstrated that the remaining LC neurons compensate for neuronal loss by increasing NE production and transmission (Szot et al., 2006). Human AD brains in Braak Stages III, IV show increased CB1 activity in hippocampal and inner frontal cortical layers (Manuel et al., 2014). Preclinical studies from our lab using the selective LC-neurotoxin DSP-4 to induce LC degeneration indicate increased indices of eCB activity. These included increases in DGL-a, MAGL and FAAH expression levels in the PFC of male rats (Urquhart et al., 2019). By the time disease progression reaches Braak stages V, VI there is human postmortem evidence for the upregulation of ARs in the PFC and hippocampus, thus suggesting that LC degeneration is causing decreases in NE transmission at this point (Kalaria et al., 1989). The loss of NE tone is supported by studies that have reported decreased concentrations of NE in the hypothalamus, cingulate, putamen and raphe nuclei (Mann et al., 1980; Arai et al., 1984). Further, during Braak stages V, VI, it has been shown that CB1r levels are decreased from their elevated state, returning to baseline levels (Manuel et al., 2014). While our hypothesis would be difficult to test directly, there is preclinical evidence that supports the use of cannabinoid interventions in early stages of disease. For example, AβPP/PS1 rodents chronically treated with CBD (50 mg/kg; daily for 3 weeks) showed reduced levels of soluble Aβ_40_ in the hippocampus and restored learning and memory in social and spatial memory assessments (Watt et al., 2020). Another study utilizing AβPP/PS1 demonstrated that chronic combined treatment with CBD and THC at early symptomatic stages reduced cortical Aβ_42_ levels and altered plaque composition, resulting in preserved memory function (Aso et al., 2015; Tables 2, 3).

**TABLE 2:** Influence of CBD/THC or non-selective agonists on neuropathology in AD transgenic rodent models.

Neuropathology	CBD	Cannabinoid system modulators (CB1 and CB2 Activators; ECB Reuptake Inhibitors, THC + CBD Combined Treatment)
**Aβ _42_ and Tau-p**	AbPP/PS1 Chronic treatment (i.p. 50 mg/kg; daily for 3 weeks): ↓ soluble Ab_40_ in hippocampus (Watt et al., 2020). Rat AD model PPARg-dependent: -Reduction in reactive gliosis and neuronal damage -Stimulated hippocampal neurogenesis (Esposito et al., 2011).	AbPP/PS1 Chronic THC + CBD Combined Treatment at early symptomatic stages: ↓Ab_42_ levels and altered plaque composition (Aso et al., 2015). Chronic THC + CBD Combined Treatment at later symptomatic stages: ↓GluR2/3 ↑ GABA _A_ Ra1 (Aso et al., 2016). Hyperglycemic 3xFAD Mice Chronic low dose AM404 (0.5uM); GSK-3b inhibitor; AEA Reuptake Inhibitor) ↓Ab_42_ levels ↓tau phosphorylation (Huang et al., 2019).
**Inflammation** **neuronal injury**	AbPP/PS1 Chronic treatment (i.p. 50 mg/kg; daily for 3 weeks): NO EFFECT on markers of inflammation, degeneration, or PPARg, in the cortex (Watt et al., 2020). 5xFAD ↓ IL-5 (Khodadadi et al., 2021).	AbPP/PS1 Chronic THC + CBD Combined Treatment at early symptomatic stages: ↓astrogliosis, microgliosis and inflammatory markers (Aso et al., 2015). ↓ Oxidative phosphorylation ↓ TNF pathways ↑Tautophagy (Hao and Feng, 2021). 5x FAD Mice FAAH Inhibitors URB597 or SR1 No beneficial effect on cytokine expression (Vazquez et al., 2015). Hyperglycemic 3xFAD Mice Chronic low dose AM404 (0.5 μM; GSK-3b inhibitor; AEA Reuptake Inhibitor) ↓astrogliosis (GFAP), microgliosis (Iba1) (Huang et al., 2019).
**Cognition/BPSD**	AbPP/PS1 Chronic treatment (i.p. 20 mg/kg; daily for 3 weeks OR i.p. 50 mg/kg; daily for 3 weeks) Reversed social recognition and novel object recognition deficits without causing anxiety-related behaviors (Cheng et al., 2014; Watt et al., 2020). 5xFAD Improved cognitive function (Morris Water Maze) Reduced anxiety (Open Field Test) (Khodadadi et al., 2021). 14 month-old Tau 58/2 mice Chronic treatment (100 mg/kg i.p. daily for 3 weeks) ↓ anxiety-like behavior ↓ contextual-fear associated freezing (Kreilaus et al., 2022).	AbPP/PS1 Chronic THC + CBD Combined Treatment at early OR late symptomatic stages: -Preserved memory -Reduced memory impairment (Aso et al., 2015). 5x FAD mice FAAH Inhibitors URB597 or SR1 No effect on Morris Water Maze (Vazquez et al., 2015). Hyperglycemic 3xFAD Mice Chronic low dose AM404 (0.5 μM; GSK-3b inhibitor; AEA Reuptake Inhibitor) Improved learning and memory deficits in Morris Water Maze (Huang et al., 2019).

**TABLE 3:** Influence of CBD/THC on *in vitro*/non-ad models of Alzheimer’s disease-related neuropathology.

Neuropathology	CBD	Cannabinoid system modulators (CB1 and CB2 Activators; ECB Reuptake Inhibitors, THC + CBD Combined Treatment)
**Aβ _42_ and Tau-p**	↓Aβ neurotoxicity *via* inhibiting caspase-3 (Haghani et al., 2012). Ab_42_ stimulated PC12 neuronal cells ↓Ab-induced tau-p ↓GSK3b ↑ Wnt/b-catenin pathway ↑ APP ubiquitination ↓Ab_42_ *Via* stimulation of PPARg (Vallee et al., 2017). ↓tau-hyper-p Rescue mediated by Wnt/b-catenin pathway (Esposito et al., 2006c). Ab_42_ stimulated PC12 neuronal cells and C6 rat glioma cells: ↓NO-dependent tau-p (Esposito et al., 2006b).	Ab_42_ stimulated PC12 neuronal cells and C6 rat glioma cells: WIN-55,212-2 ↓NO-dependent tau-phosphorylation (Esposito et al., 2006a). Ab_42_ stimulated hippocampal neuronal and glial co-cultures: URB 602 or JZL184; MAGL Inhibitors ↑s-AG ↓ Ab_42_-induced neurodegeneration and apoptosis ↓ ERK1/2, NFkb phosphorylation (Chen et al., 2011). Traumatic brain injury PF04457845 (FAAH Inhibitor) ↑)EA ↓tau phosphorylation ↓GSK-3b phosphorylation ↓p35/25 (caspase cleavage products) (Selvaraj et al., 2019).
**Neuroinflammation, neuronal injury**	↓ Inflammation ↓ GFAP and iNOS expression ↓ nitrites, and IL-1b (Esposito et al., 2007a). ↓ reactive gliosis ↓ neuronal damage Effects dependent on PPARg interaction (Esposito et al., 2011). Ab_42_ stimulated PC12 neuronal cells and C6 rat glioma cells: ↓NO-dependent tau-phosphorylation ↓ nitrite production ↓iNOS expression Inhibition of p-38 Inhibition of MAPK Inhibition of NFkB (Esposito et al., 2006b). Ab_42_ stimulated PC12 neuronal cells ↓oxidative stress ↓mitochondrial dysfunction ↓Reactive Oxygen Species (ROS) (Vallee et al., 2017).	Ab_42_ stimulated PC12 neuronal cells and C6 rat glioma cells: WIN-55,212-2 ↓ NO and iNOS expression (Esposito et al., 2006a). Prevents inflammatory activation, loss of neuronal markers, and cognitive deficits in Aβ-treated rats (Ramírez et al., 2005). Traumatic brain injury Cannabinoids, *via* activation of CB1R/CB2R are: -neuroprotective against excitotoxicity and acute brain damage both *in vitro* and *in vivo* -*via* blockade of excitotoxicity -reduction of calcium influx -antioxidant properties -enhanced trophic factor support (van der Stelt et al., 2002; Mechoulam and Shohami, 2007). PF04457845 (FAAH Inhibitor) ↑)EA ↓IL1-b, IL-6 and TNF-a (Selvaraj et al., 2019).

(Ramírez et al., 2005; Esposito et al., 2006a,b,c, 2007a,b, 2011; van der Stelt et al., 2006; Chen et al., 2011; Vallee et al., 2017; Selvaraj et al., 2019).

#### Targeting cannabinoid cell signaling to preserve cell viability in vulnerable stress responsive nuclei

Alongside the normally fluctuating and reversable influence of glucocorticoid-mediated changes in mPFC dendritic spine density, we have proposed that chronic stress results in the production and accumulation of Aβ in the PFC, initiating mechanisms of post synaptic depression (Ross et al., 2018; Figure 2). Over time, and with aging, the combined effect of reduced neuronal resilience to stress- and glucocorticoid-mediated changes in PFC structure and plasticity, and the accumulation of Aβ, with subsequent development of senile plaques and neurofibrillary tangles has synergistic potential for cognitive decline. In line with this rationale and that of the previous section on circuitry, a collective inhibition or down-regulation of activity in the mPFC and concurrent increase in activity of the amygdala may serve as a mechanism by which the modulation and organization of stress integrative circuitry is altered under conditions of chronic stress. In this section, we delve deeper into mechanisms of synaptic plasticity, focusing on the cell signaling mechanisms that foster or inhibit dendrite growth and complexity. Further, we compare the cell signaling pathways of stress mediators with those of the cannabinoid system to better understand the therapeutic potential of cannabinoids in counteracting the deleterious effects of stress in AD (Figures 4, 6).

Preclinical studies using the chronic restraint stress paradigm for 21 days demonstrated regionally specific functional and structural changes in the PFC, amygdala, and hippocampus (McEwen and Gianaros, 2011). Several studies have demonstrated that chronic stress results in a reduction (Cook and Wellman, 2004; Radley et al., 2004; Goldwater et al., 2009) or loss of apical dendritic spine length in pyramidal neurons of the mPFC (Hill et al., 2011b; McEwen and Morrison, 2013). Pyramidal neurons in layer III of the infralimbic, prelimbic, and cingulate cortices are reduced by 20% an effect downstream of protein kinase C (PKC) signaling (Hains et al., 2009). This dendritic shrinkage was accompanied by spine over 30% spine loss, which were primarily thin spines that are known to be involved in plasticity and cognitive performance, following chronic stress (Bloss et al., 2011). While dendritic spines of the mPFC are reduced following chronic stress (Wellman, 2001; Cook and Wellman, 2004; Radley et al., 2004, 2006b; Cerqueira et al., 2005, 2007a,b), it has been demonstrated that dendritic spines in the amygdala grow longer and more complex (Vyas et al., 2006). Moreover, studies of repeated stress in rodents have shown hyper-excitability of BLA neurons (Shekhar et al., 2005; Vyas et al., 2006). Thus, it is possible that the hyperactivation of the BLA afferents to the PFC weaken cognitive function, effectively shifting to increased amygdalar processing in AD (McLaughlin et al., 2009; Figure 2). Several preclinical studies indicate therapeutic potential for cannabinoids in treating stress-induced PFC dysfunction (Tables 2–5). For example, CB_1_r knockout mice exposed to chronic restraint stress exhibit enhanced dendritic arborization in the BLA and concurrent reciprocal dendritic shrinkage in the PFC (Hill et al., 2011a). Conversely, FAAH knockout mice were found to have elevated dendritic arborization of BLA neurons, thought to arise from AEA signaling (Hill et al., 2013; Tables 4, 5). A study using FAAH inhibitors in the PFC revealed an increase in PFC dendrite volume (Hermes et al., 2018).

**TABLE 4:** Distinct contributions of CB1R or CB2R on stress and neuropathology in AD transgenic rodent models.

Neuropathology	Selective CB1 agonist/CB1-dependent effects	Selective CB2 agonist/CB2-dependent effects
**Aβ _42_ and Tau-p**	APP/PS1xCB1-KO (heterozygous) -NO EFFECT on Ab density or plaque deposition ↓ PSD-95 Expression (Aso et al., 2018).	AbPP/PS1 JWH-133 Treatment at pre-symptomatic stages: ↓ tau phosphorylation at Th181 -NO EFFECT on Ab_42_ production/deposition in cortex or hippocampus (Aso et al., 2013). APP/PS1xCB2-KO (HOMOZYGOUS) ↑O (HOMOZ Ab_42_ deposition ↑deposit Ab_40_ (Aso et al., 2016).
**Inflammation, neuronal injury**	APP/PS1xCB1-KO (homozygous) -CB1-KO drastically reduced survival of APP/PS1 Mice. (Aso et al., 2018).	AbPP/PS1 JWH-133 Treatment at pre-symptomatic stages: ↓microglial reactivity ↓proinflammatory cytokine expression (IL-1b, IL-6, TNF-a, IFNg) ↑ INACTIVE GSK3b ↓ active p38, SAPK/JNK (Aso et al., 2013). APP/PS1xCB2-KO (homozygous) NO EFFECT on viability of APP/PS1 Mice (Aso et al., 2016).
**Cognition/BPSD**	APP/PS1xCB1-KO (heterozygous) Accelerated memory impairment (Aso et al., 2018).	AbPP/PS1 JWH-133 Treatment at pre-symptomatic stages: Enhanced learning and memory (Aso et al., 2013) APP/PS1xCB2-KO (HOMOZYGOUS) -Attenuated the beneficial cognitive effects of cannabis (Aso et al., 2016).

**TABLE 5:** Distinct contributions of CB1R or CB2R on *in vitro*/non-AD models of Alzheimer’s disease-related neuropathology.

Neuropathology	Selective CB1 agonist/CB1-dependent effects	Selective CB2 agonist/CB2-dependent effects
**Aβ_42_ and Tau-p**	Arachidonyl-2′-chloroethylamide (ACEA) co-treatment with Aβ_42_ PREVENTED -Aβ_42–_-induced changes (↓) in evoked neuronal activity (Haghani et al., 2012). Aβ_42_ stimulated PC12 neuronal cells and C6 rat glioma cells: ACEA Treatment **↓NO-dependent tau-p** (. Esposito et al., 2006a). SH-SY5Y neuroblastoma cells: mRVD-hemopressin (RVD) REVERSED -Apoptosis -Suppression of neurite outgrowth -Suppression of PSD95 -*Via* INHIBITION of PKA and GSK3b -Effects ablated with CB1 antagonist (Zhang R. et al., 2020). *In Vivo* STZ exposure *In Vitro* STZ exposure ACEA ACEA -Reversed Cognitive impairment Modulates STZ-induced NO release ↑ndu-induced Akt/ERK Pathways Rescues cell death ↑ anti-apoptotic proteins (Bcl-2) (Crunfli et al., 2019).	CB2R knockout mice -AD-like tau hyperphosphorylation, **↑** GSK3β activity ↓ of AMPK activity ↓Sirt1 activity ↓mitochondria dysfunction (Wang et al., 2018). HEK293 tau cells (JWH133) ↓ reduces phosphorylation of tau ↓GSK3β activity AMPK-dependent (Wang et al., 2018).
**Neuroinflammation, neuronal injury**	**↓** Aβ_42_-induced reactive gliosis (Esposito et al., 2007a,2006b). Aβ_42_ stimulated PC12 neuronal cells and C6 rat glioma cells: ACEA treatment **↓ NO and iNOS expression** (Esposito et al., 2006a).	
**Cognition/BPSD**	Rescued memory to baseline after the bilateral Administration of Aβ peptides into the PFC of adult rat (Haghani et al., 2012).	CB2R knockout mice -Hippocampus-dependent memory impairment (Wang et al., 2018).

As discussed earlier, studies using CRF OE mice show increased levels of tau-p at the AD relevant AT8, PHF-1 and S422 sites compared with wild type littermates. The effects at the AT8 and PHF-1 sites were found to be CRFR1 dependent, as chronic treatment for 30 days with a selective CRFR1 antagonist (30 days; R121919), completely prevented the rise in hyperphosphorylated tau (Figure 3). CRF OE rodents broadly show increased levels of phosphorylated GSK-3β, mitogen-activated protein kinases (MAPK) p38, and extracellular signal-regulated kinase (ERK)1/2. Levels of cyclin-dependent kinase 5 (cdk5) were unchanged in CRF OE mice regardless of treatment with R12919. However, the only cell signaling pathway found to be CRFR1 dependent was that of the c-Jun N- terminal kinase (JNK) indicating that the induction of tau-p *via* JNK activation is central to tau-p at the AT8 and PHF-1 sites in CRF OE (Campbell et al., 2015a,b). This holds additional significance when considering the treatment potential of cannabinoids for the reversal of stress-related neuropathy as pretreatment of AβPP/PS1 mice with the selective CB2 agonist JWH-133 at early stages of disease reduced active p38, and JNK while increasing levels of inactive GSK3β *in vivo* (Aso et al., 2013) and *in vitro* (Wang et al., 2018), effects that are thought to be dependent on Wnt7a signaling (Ciani et al., 2011; Figure 6).

#### Harnessing cannabinoid influence on widespread neuroimmune responses

A recent meta-analysis indicates that markers of microglial activation are consistently elevated in postmortem brain tissue from AD patients compared to non-neurological age-matched controls (Hopperton et al., 2018). Several studies included a “high pathology” cohort of individuals that were cognitively normal (no impairment), but exhibited high levels of AD-related neuropathology. Markers of neuroinflammation were significantly higher in cognitively impaired AD patients compared to their high pathology counterparts (Hopperton et al., 2018). These data support the increasingly recognized notion that inflammation is a driving factor of AD progression, rather than a mere side effect of neurodegenerative processes (Gomez-Nicola and Boche, 2015).

**FIGURE 7:**
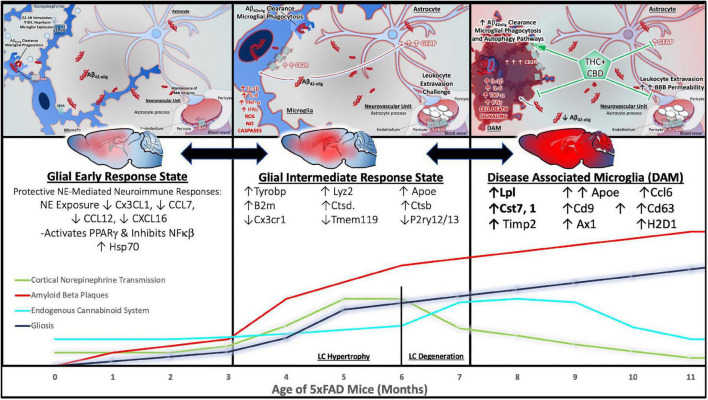
Inflammatory progression in 5xFAD AD mouse model and beneficial effects of cannabinoids. The time course of neuropathology and inflammation in the 5xFAD model of AD LC-NE modulation of inflammation, LC degeneration, and the development of degeneration-associated microglial (DAM) phenotypes that have been defined by single-cell RNA polymerase experiments. 5xFAD mice develop cerebral amyloid plaques and gliosis by 2 months, exhibit reduced synaptic markers, neuronal loss and memory impairment by 4 months, and continue to accumulate extra- and intraneuronal Aβ_42_ between 12 and 16 months of age. While intact, the LC-NE system is neuroprotective. NE mediates an increase in protein expression of the 70 kilodalton heat shock protein 70 (hsp70), and a reduction of nitric oxide synthase (NOS). By 3 months, 5xFAD mice show a significant increase in CB2r (Panel 1, left). By 5 months there is significant evidence of LC damage and inflammation. At 6 months, studies have demonstrated a shift in microglia phenotype from a homeostatic or early response state to an intermediate response state, and this transition is defined by the upregulation of AD-associated genes (B2m,Ctsd,Ctsb, Fth1, Tyrobp and apoe) and concurrent downregulation of the homeostatic CxCL1, P2ry12/P2ry13, and Tmem119 genes (Panel 2, center). 5xFAD mice show intrinsically decreased CB1r, decreased MAGL, increased DAGL and increased CB2r expression at 11 months. Studies evaluating microglial phenotypes in 5xFAD mice at 8 months show a distinct, TREM-2 dependent transition to the late response state known as DAM. The DAM phenotype is characterized by an increase in AD associated genes such as Lpl, apoe, and the TREM2-Tyrobp signaling complex. Importantly, a recent study demonstrates that in the 5xFAD model, CBD could improve the behavioral and cognitive function of AD mice by modulating the expression of IL-5 (Khodadadi et al., 2021). Other mechanisms of CBD + THC mediated protection include the reduction of inflammatory cytokines, promoting autophagy and microglial phagocytosis of amyloid plaques and maintaining blood brain barrier integrity by preventing leukocyte extravasion (Panel 3, right).

In addition to playing a central role in mediating and coordinating stress responses, the LC-NE system plays a supportive role in modulating neuroinflammation (Heneka et al., 2015; Ross et al., 2015). It has been suggested that LC neuronal loss and consequent changes in NE transmission render the brain more vulnerable to inflammatory insults. Moreover, LC-NE depletion can contribute to increased Aβ plaque burden (Kalinin et al., 2007). In this regard, the timing of an effective intervention will be critical to preserving LC-NE system integrity, thereby reducing the negative inflammatory and degenerative sequalae that follow LC neuronal injury. Figure 7 depicts the time course of the 5xFAD model of AD in terms of its well-characterized AD pathology (Oakley et al., 2006), LC-NE modulation of inflammation, LC degeneration (Kalinin et al., 2012), and the development of degeneration-associated microglial (DAM) phenotypes that have been defined by single-cell RNA polymerase experiments (Keren-Shaul et al., 2017; Mathys et al., 2017). 5xFAD mice develop cerebral amyloid plaques and gliosis by 2 months, exhibit reduced synaptic markers, neuronal loss and memory impairment by 4 months, and continue to accumulate extra- and intraneuronal Aβ_42_ well into the range of 2–10 ng per mg total protein between 12 and 16 months of age (Oakley et al., 2006). The investigation of the LC-NE system in the first 3 months of 5x FAD mice reveal that while intact, the LC-NE system acts in a neuroprotective manner against the growing Aβ_42_ burden by increasing protein expression of the 70 kilodalton heat shock protein 70 (hsp70), reducing nitric oxide synthase (NOS) expression and increasing the expression of CCL2, also known as MCP-1 (Heneka et al., 2003). Other studies have indicated that by 3 months 5xFAD mice show a significant increase in CB2r (Lopez et al., 2018). By 5 months there is significant evidence of LC damage and inflammation (Kalinin et al., 2012), and 1 month of treatment with the synthetic NE precursor, L-DOPS, reduces amyloid burnden, astrocyte activation, and increased the expression of neurotrophins including BDNF, ultimately improving performance on the Morris water maze test (Kalinin et al., 2012). Around this time (6 months), other studies have demonstrated a shift in microglia phenotype from a homeostatic or early response state to an intermediate response state, and this transition is defined by the upregulation of AD-associated genes (B2m,Ctsd,Ctsb, Fth1, Tyrobp and apoe) and concurrent downregulation of the homeostatic CxCL1, P2ry12/P2ry13, and Tmem119 genes (Keren-Shaul et al., 2017). Upon LPS or Aβ_42_ challenge, NE exposure has been shown to promote microglial activation, indicated by a reduction in homeostatic gene expression (Cx3CL1, CCL7, CCL12, and CxCL16), activation of the PPARγ pathway (Klotz et al., 2003), and inhibition of NFκβ and its downstream proinflammatory targets (Heneka et al., 2015). While Keren-Shaul and colleagues did not identify the factor that led to this initial transition, termed step 1 in their framework, it is an intriguing parallel that warrants further investigation.

It is under conditions of extreme cellular stress such as these, modeled in other studies using LC-selective pharmacological lesions, that promotes the onset of LC degeneration, ultimately resulting in the loss of NE neuroprotective influence over inflammatory processes (Heneka et al., 2015). As mentioned earlier, our lab has shown increased indices of endogenous cannabinoid function in the cortex under conditions of LC degeneration and 70% loss of NE tone. We observed increased protein expression of FAAH, MGL, and DGL, suggesting increased turnover of 2-AG and possibly an increase or extension of AEA activity. The only study examining the intrinsic ECB system of 5xFAD mice show decreased CB1r, decreased MAGL, increased DAGL and increased CB2r expression at 11 months. Thus, based on the available data, it cannot be definitely stated if the ECB system compensates for loss of neuroprotective NE immunomodulation at this critical window (6 months). Studies evaluating microglial phenotypes in 5xFAD mice at 8 months show a distinct, TREM-2 dependent transition to the late response state known as DAM (Keren-Shaul et al., 2017). The DAM phenotype is characterized by an increase in AD associated genes such as Lpl, apoe, and the TREM2-Tyrobp signaling complex (Keren-Shaul et al., 2017). Importantly, a recent study demonstrates that in the 5xFAD model, CBD could improve the behavioral and cognitive function of AD mice by modulating the expression of IL-5 (Khodadadi et al., 2021). In contrast, THC administration in 5xFAD mice at 4 months old, induced COX-2 activation, resulting in synaptic and memory impairments. The neuroprotective effects of THC could be rescued by COX-2 inhibition (Chen et al., 2013).

Several other genetically modified models of AD have been utilized widely for the study of cannabinoid and immune interactions in the context of well-established pathological features of AD. Amongst the most widely used, the AβPP/PS1 mouse line has been subjected to several cannabinoid modulating conditions (Aso et al., 2015, 2016, 2018) and drugs at various doses and time courses (Cheng et al., 2014; Aso et al., 2015, 2016; Aso and Ferrer, 2016; Watt et al., 2020; Hao and Feng, 2021; see Tables 2, 4). The beneficial effects of cannabis interventions, particularly chronic administration of combined THC + CBD, are evident as reduced Aβ_42_ plaque burden and altered composition, reduced inflammatory markers, gliosis (Aso et al., 2015), oxidative phosphorylation, downregulation of TNF pathways and upregulation of autophagy (Hao and Feng, 2021). Consequently, THC + CBD treatment of AbPP/PS1 mice has demonstrated the ability to preserve and reduce impairment of memory, as well as reverse social and novel object recognition deficits (Cheng et al., 2014; Aso et al., 2015; Watt et al., 2020). *In vitro* and *in vivo* models of Aβ_42_-induced neuronal injury, inflammation, and tau-phosphorylation have also been studied using cannabinoid agonists, demonstrating that CBD and other cannabinoid system modulators are capable of reversing these deleterious effects, preventing the induction of inflammatory cascades (Ramírez et al., 2005; Esposito et al., 2006a,b,c, 2007a,b, 2011; Haghani et al., 2012; Vallee et al., 2017; see Table 3) and protecting against excitotoxicity (van der Stelt et al., 2006; Mechoulam and Shohami, 2007).

The individual contributions of CB1r and CB2r in the context of AD neuropathology have also been extensively studied in AD transgenic mice (see Table 4), or conditions of Aβ_42_-induced pathology *in vitro* and *in vivo* (Esposito et al., 2006a,2007a,b; Haghani et al., 2012; Wang et al., 2018; Zhang R. et al., 2020; see Table 5), but the synergism of CB1r and CB2r activation (*via* THC) in the presence of a negative allostatic modulator (CBD) appears to demonstrate the most favorable therapeutic profile (Aso et al., 2015; Aso and Ferrer, 2016; Schubert et al., 2019). The therapeutic potential of targeting CB2r alone has been explored and may hold potential for earlier stages of intervention to facilitate Aβ_42_ clearance, as CB_2_r regulates inflammatory processes such as the release of cytokines and leukocyte extravasation (Ramirez et al., 2012; Cabanero et al., 2020). It is important to note that the presence of CB_2_r in the brain is typically indicative of brain injury or neurodegenerative disease (Donat et al., 2014; Braun et al., 2018), and postmortem AD brains express an abundance of CB_2_r. CB_2_ agonists facilitated the reduction of Aβ plaques in human AD brain tissue and *in vivo* macrophage phagocytosis of Aβ fibrils (Tolon et al., 2009). Treatment of the AβPP/PS1 rodent model with the selective CB2r agonist, JWH-133 at pre-symptomatic stages effectively reduced microglial reactivity, the expression of proinflammatory cytokines including IL-1β, IL-6, TNF-α, and IFNγ. The cell signaling modulators thought to be downstream of this CB2r mediated effect are GSK-3β, which was found to be present in lower amounts, reduced p38 and JNK pathways. Consequently, learning and memory was improved in JWH-133 treated AβPP/PS1 rodents (Aso et al., 2013). These CB2r-dependent effects were also observed *in vitro* alongside reduced phosphorylation of tau and GSK-3β activity and were abrogated *in vivo* in rodents null for CB2r (Wang et al., 2018). However, it has been demonstrated that a superior effect is obtained when using combined THC and CBD treatments at early symptomatic stages in the AβPP/PS1 model. Chronic THC + CBD treatment reduced astrogliosis and microgliosis, inflammatory markers (Aso et al., 2015), oxidative phosphorylation, TNF pathways and increased autophagy (Hao and Feng, 2021; Tables 2–5).

Human studies on the effects of psychological and life stress on the inflammatory response have identified vulnerable populations that are susceptible to higher stress responses of inflammatory mediators (see Segerstrom and Miller, 2004; Rohleder, 2019 for full review). These include individuals with lower socioeconomic status (Steptoe et al., 2002; Brydon et al., 2004, 2012), and those with higher work stress associated with effort-reward imbalances (Hamer et al., 2006). Additionally, other indices of overall wellness and the impact of stress in older adults, such as poor sleep were reported with higher levels of IL-6 inflammatory responses (Heffner et al., 2012). Moreover, inflammatory responses were found to be related to acute stress responses, including higher levels of inflammatory mediators in individuals with higher states of anger and anxiety (Carroll et al., 2011). Amongst the available data, it appears that IL-6 and IL-1β are the most salient inflammatory mediators influenced by psychological life stress (Yamakawa et al., 2009; Aschbacher et al., 2012; Puterman et al., 2014), consistent with the putative role of CRF signaling (Jain et al., 1991; Saperstein et al., 1992). This evidence is congruent with behavioral and molecular studies that have demonstrated the ability of CB_1_ agonists to reduce inflammation (Crunfli et al., 2019) and Aβ neurotoxicity by inhibiting caspase-3 (Haghani et al., 2012), an apoptotic signaling molecule associated with the CRFR1-PKC pathway that is activated under conditions of chronic stress (Lichlyter et al., 2022).

## Conclusion

In this review we have focused on the mechanisms by which the cannabinoid system can potentially limit hyperactivity of stress neurocircuitry, and in doing so, have discussed the potentially protective or anti-stress features that may benefit AD patient populations. There are several other potential beneficial aspects of cannabis for the treatment of AD that are outside the scope of this review. We direct the reader to an excellent review on the available preclinical data that support the therapeutic potential of cannabinoids in treating AD (Aso and Ferrer, 2014).

Our aim was to highlight the significance of the eCB as a mediator of the stress response that has therapeutic potential in reversing or halting the deleterious effects of stress that contribute to AD neuropathology. The eCB/cannabinoid receptor signaling is capable of adapting eCB tone during acute and chronic stress throughout the central nervous system and peripheral nervous system. We have illustrated how, under conditions of allostatic overload/chronic stress conditions, elevated tone of stress mediators such as NE, CRF and can become detrimental. We have discussed specific brain regions (mainly LC, PFC, and amygdala) implicated in AD pathology that may be exacerbated by stress. This approach offers a different perspective of AD hallmark by incorporating the “prion-like hypothesis” while not mutually excluding the amyloid cascade hypothesis. We believe that the LC-NE dysregulation is plausible given the centrality of the LC within the coeruleo-cortico-amygdalar circuit and early Braak staging of NFTs in the LC and limbic region. Furthermore, emphasizing the concept of allostatic overload in translational research and medicine may highlight non-memory symptoms and asymptomatic AD individuals that are often excluded from the clinical picture.

The synergistic interaction of eCBs along with the compensatory effects of the coeruleo-cortico-amygdalar circuit under allostatic overload/chronic stress is an area of interest that is relevant for AD-related research as well as clinical populations. Effectively harnessing the eCB system has the potential to restore allostatic balance, thereby influencing cognition, behavior, autonomic processes, endocrine function, and neuroplasticity. The knowledge of this eCB homeostatic paradigm may be utilized as a preventative measure before maladaptive LC-NE dysregulation and a disease modifying therapy for AD. Premature measures to capitalize on eCB buffering prolonged noradrenergic tone and release before AD neuropathology ensues is essential. Moreover, the complexity of interactions between stress and AD neuropathology may become integrated into a feed-forward system that ultimately leads to neurodegeneration. When considering AD related pathology that may originate as chronic responses to stress or may simply be aggravated by stress in later disease stages, there is compelling evidence for the use of combined THC + CBD in formulations such as Sativex. Further investigations should examine the tiered eCB capability to rebound from progressive proteinopathy-induced LC-NE dysfunction. Additionally, future goals should be directed toward redefining parameters that consider the heterogeneity of AD such as the preclinical and prodromal stages of AD. Moreover, stratifying patients based on Braak staging and obtaining a holistic psychological history including the presence of trauma and/or chronic stress could be beneficial in determining if cannabis is likely to be an effective treatment for a particular individual.

## Author contributions

RS conducted the literature search, summarized the currently available research on the subject, and co-wrote the manuscript. JR co-wrote the manuscript, created the figures and tables, and reviewed the interpretation of the data described in the manuscript. EV provided the funding to make this work possible and reviewed the manuscript prior to submission. All authors contributed to the article and approved the submitted version.

## Abbreviations

ACEA, Arachidonyl-2 ′ -chloroethylamide: 11
ACEA action, synthetic CB1r selective agonist: 11
ACTH, adrenocorticotropic hormone: 5
AD, Alzheimer’s Disease: 3
APOE, apolipoprotein E: 3
APP, amyloid precursor protein: 3
APP/PS1, amyloid precursor protein/presenilin 1: 9
APPswe/PS1dE9, amyloid precursor protein/presenilin 1 double transgenic: 9
AR, adrenergic receptor: 5
A β, Amyloid Beta Peptide: 4
BBB, blood brain barrier: 5
BPSD, behavioral and psychological symptoms of dementia: 4
CBD, Cannabidiol: 11
CBD action, Negative Allosteric Modulator of CB1: 11
cdk-5, cyclin-dependent kinase 5: 16
CeA, central nucleus of the amygdala: 6
CRF, corticotropin releasing factor: 5
CRF-OE, overexpress CRF: 7
CRFR1, CRF receptor 1: 7
DSM-5, Diagnostic and Statistical Manual of Mental Disorders 5: 3
eCBRIs, endocannabinoid reuptake inhibitors: 11
ELISA, enzyme linked immunoassay: 7
ERK1/2, extracellular signal-regulated kinase: 16
GFAP, glial fibrillary acidic protein: 7
GSK3 β, glycogen synthase kinase 3 beta: 5
HPA, hypothalamic-pituitary-adrenal: 3
IL-mPFC, infralimbic mPFC: 6
JNK, c-Jun N- terminal kinase: 16
JWH-133, Synthetic CB2r Selective Agonist: 11
LC, locus coeruleus: 6
LOAD, late onset AD: 3
MAPK, mitogen-activated protein kinases: 16
MMSE, Mini Mental State Examination: 8
NE, norepinephrine: 6
NFT, neurofibrillary tangles: 5
NIA-AA, National Institute of Aging/Alzheimer’s Association: 3
NINCDS-ADRDA, National Institute of Neurological and Communicative Diseases and Stroke/Alzheimer’s Disease and Related Disorders Association: 3
NO, nitric oxide: 5
PET, positron emission tomography: 4
PFC, prefrontal cortex: 6
PL-mPFC, prelimbic medial PFC: 6
PSEN1, presenilin 1: 3
PSEN2, presenilin 2: 3
PVN, paraventricular nucleus of the hypothalamus: 5
VSMCs, vascular smooth muscle cells: 5
WIN 55-212, 2, Non-selective (CB1 and CB2) Cannabinoid Agonist: 11
β -APPs, β -amyloid precursor proteins: 8.
